# Identification of cell type-specific methylation signals in bulk whole genome bisulfite sequencing data

**DOI:** 10.1186/s13059-020-02065-5

**Published:** 2020-07-01

**Authors:** C. Anthony Scott, Jack D. Duryea, Harry MacKay, Maria S. Baker, Eleonora Laritsky, Chathura J. Gunasekara, Cristian Coarfa, Robert A. Waterland

**Affiliations:** 1 Department of Pediatrics, Baylor College of Medicine, USDA/ARS Children’s Nutrition Research Center, Houston, TX USA; 2 Department of Molecular & Cell Biology, Baylor College of Medicine, Houston, TX USA; 3 Dan L. Duncan Comprehensive Cancer Center, Baylor College of Medicine, Houston, TX USA; 4 Department of Molecular & Human Genetics, Baylor College of Medicine, Houston, TX USA

**Keywords:** DNA methylation, Read-level, WGBS, Imputation, Machine learning, Random forests, Deconvolution, Bisulfite-seq

## Abstract

**Background:**

The traditional approach to studying the epigenetic mechanism CpG methylation in tissue samples is to identify regions of concordant differential methylation spanning multiple CpG sites (differentially methylated regions). Variation limited to single or small numbers of CpGs has been assumed to reflect stochastic processes. To test this, we developed software, Cluster-Based analysis of CpG methylation (CluBCpG), and explored variation in read-level CpG methylation patterns in whole genome bisulfite sequencing data.

**Results:**

Analysis of both human and mouse whole genome bisulfite sequencing datasets reveals read-level signatures associated with cell type and cell type-specific biological processes. These signatures, which are mostly orthogonal to classical differentially methylated regions, are enriched at cell type-specific enhancers and allow estimation of proportional cell composition in synthetic mixtures and improved prediction of gene expression. In tandem, we developed a machine learning algorithm, Precise Read-Level Imputation of Methylation (PReLIM), to increase coverage of existing whole genome bisulfite sequencing datasets by imputing CpG methylation states on individual sequencing reads. PReLIM both improves CluBCpG coverage and performance and enables identification of novel differentially methylated regions, which we independently validate.

**Conclusions:**

Our data indicate that, rather than stochastic variation, read-level CpG methylation patterns in tissue whole genome bisulfite sequencing libraries reflect cell type. Accordingly, these new computational tools should lead to an improved understanding of epigenetic regulation by DNA methylation.

## Background

DNA methylation, which occurs predominantly at CpG dinucleotides in mammals, is a stable epigenetic mechanism that regulates cell type-specific gene expression [1]. Accordingly, different cell types exhibit differential DNA methylation at many genomic regions [2–5]. Whole genome bisulfite sequencing (WGBS), the current gold standard for the study of DNA methylation, provides single-nucleotide resolution at all cytosines in the genome. The mean methylation level is typically reported at each cytosine, but in bulk tissues comprised of multiple cell types, this value is a weighted average across all cells in the sample, obscuring cell type-specific differences. While single-cell WGBS theoretically overcomes this problem [6, 7], technical and practical limitations result in low mapping efficiencies and poor genomic coverage, typically less than 0.5X [8, 9].

Computational solutions to identify cell type-specific signals within bulk WGBS data would offer two major advantages: high genomic coverage and applicability to existing datasets. Previous approaches to quantify read-level heterogeneity in WGBS data involved the development of various metrics, each yielding a single numeric value for any genomic region. Epipolymorphism [5] quantifies the proportion of distinct read-level patterns of methylation (referred to as epi-haplotypes) by calculating the probability that two randomly sampled reads contain different epi-haplotypes. Another metric, methylation entropy [10], assesses read-level heterogeneity by an “information-theoretic” approach based on Shannon’s entropy. Neither of these techniques, however, considers patterns of co-methylation within reads. Guo et al. [11] introduced methylation haplotype load (MHL) to quantify the fraction of fully methylated haplotypes of all lengths. Although a potential improvement over epipolymorphism and methylation entropy due to its ability to distinguish different combinations of methylation haplotypes, MHL fails to distinguish cell type-specific methylation patterns in certain contexts (Supplementary Figure 1). Importantly, none of these metrics captures the numbers, proportions, or specific patterns of unique epi-haplotypes. Another method, epiG [12], does utilize methylation patterns in WGBS reads, but identifies only one dominant epi-haplotype in each sample, essentially disregarding cell type heterogeneity. Other attempts at exploring read-level information in WGBS data sets have focused on minority regions such as those exhibiting “bipolar methylation” [13] or containing “hypo-methylated alleles” [14], rather than assessing the full breadth and depth of potential cell type-specific signals.

To overcome these limitations, we developed a new analytical approach to explore the information content of WGBS read-level heterogeneity. Our approach identifies read-level clusters of methylation patterns which associate with cell type-specific biological processes. We demonstrate the cell type specificity of these patterns by utilizing them to estimate proportions within synthetic mixtures of cells and improve the performance of methylation-based gene expression predictions. A requirement of any read-level analysis (including ours) is adequate genomic coverage of fully informative reads. We therefore developed an ancillary machine learning tool to accurately impute “missing” CpG methylation values at the read level and show that this not only improves cluster-level analysis but also substantially increases the information yield from existing WGBS datasets.

## Results

### A new approach for identifying read-level DNA methylation patterns within WGBS data

**Fig. 1:**
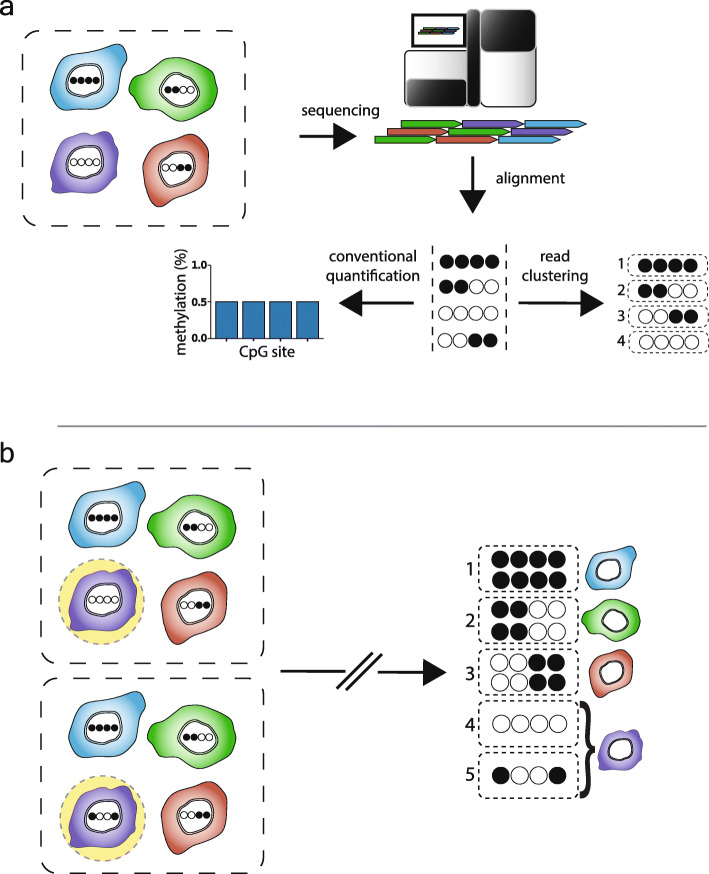
Rationale behind Cluster-Based analysis of CpG methylation (CluBCpG). **a** Each WGBS read originates from a DNA molecule within a single cell (filled and empty circles in tanghulu plots represent methylated and unmethylated CpG sites; columns and rows represent CpG sites and WGBS reads, respectively). The dotted-outline box represents a tissue sample, and colored shapes represent different cell types. Conventionally, methylation is measured by averaging methylated and unmethylated reads at each CpG site. Instead, CluBCpG groups reads based on methylation patterns. (Note: By default, 4 reads of identical methylation pattern are required to comprise a cluster; single-read “clusters” are depicted here for simplicity.) **b** Conceptually, CluBCpG can be utilized to compare two samples (dotted boxes) to find cell type-specific differences by identifying patterns that are unique to one of the input samples

We developed a software package called Cluster-Based analysis of CpG methylation (CluBCpG). CluBCpG operates on the BAM files generated by mapping WGBS reads with Bismark [15] and standard preprocessing tools (see the “Methods” section). CluBCpG segments the genome into 100-bp non-overlapping bins [16]; analysis is restricted to all such bins containing ≥ 2 CpGs (hereafter referred to as “bins”). The analysis considers only reads that overlap all CpG sites within each bin (informative reads). Reads of identical methylation patterns are grouped into “clusters” (Fig. 1a). CluBCpG is configured to operate on one or two aligned BAM files (WGBS libraries). In the two-library (comparative) analysis, reads are indexed by library then combined, enabling the identification of both shared and sample-specific clusters (Fig. 1b). CluBCpG produces a genome-wide summary of read clusters annotated by bin and detailing genomic location, methylation patterns, number of reads, and sample of origin. All analyses described herein are based on default settings, evaluating bins covered by at least 10 informative reads per library, and requiring at least 4 reads of identical methylation pattern to comprise a cluster (*P* < 0.01, *χ*^2^ test). However, CluBCpG allows these settings to be adjusted by the user.

### CpG read clusters provide cell type-specific information

**Fig. 2:**
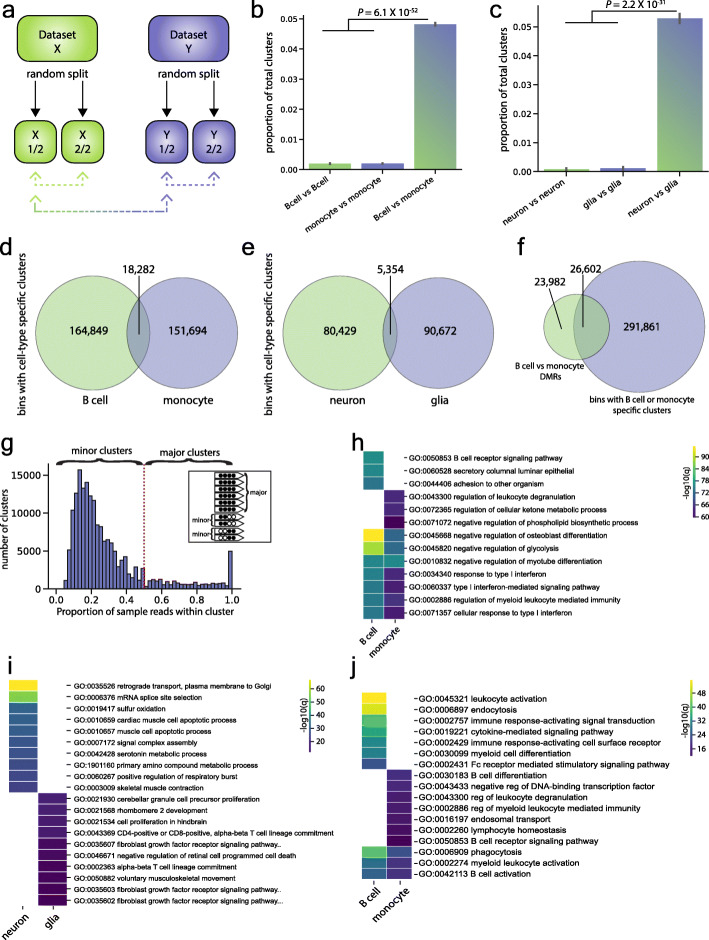
CluBCpG identifies unique read clusters that associate with cell type. **a** Schematic depicting how data were iteratively divided into random splits to perform cell type comparisons using CluBCpG. **b**, **c** Bar graphs representing the average proportion of clusters unique to either input across 10 rounds of random sampling; comparisons were performed for **b** human B cells and monocytes and **c** human neurons and glia. Error bars represent the standard deviation from the mean; statistical test: one-way ANOVA, f-statistics are 83,978 (**b**) and 6725 (**c**), 2 degrees of freedom. In both cases, > 20-fold more unique clusters were identified when different cell types are compared. **d**, **e** Venn diagrams of all genomic bins with a cell type-specific cluster identified in **d** the full data set B cell vs. monocyte comparison and **e** the neuron vs. glia comparison. **f** In the B cell vs. monocyte comparison, < 10% of bins with a cell type-specific cluster overlap with a B cell vs. monocyte DMR. **g** Histogram showing the proportional representation of sample reads per B cell-specific cluster in the B cell vs. monocyte comparison. Clusters comprising ≥ 50% or < 50% of the B cell reads in that bin are termed “major” and “minor” clusters, respectively. Inset illustrates the concept. **h**, **i** Heatmaps showing the top 10 GO biological process terms associated with bins containing **h** a B cell- or monocyte-specific cluster or **i** a neuron- or glia-specific cluster. **j** Heatmap of the top 10 GO biological process terms from B cell and monocyte bins containing a major cluster. Colors in all heatmaps represent the -log10 of the *q* value calculated by GREAT

Because each read originates from a DNA molecule in one cell, we hypothesized that read clusters carry cell type-specific information. To test this, we employed CluBCpG in the two-library mode to compare publicly available WGBS data of human B cells and monocytes (from ENCODE), each containing 30X genome-wide coverage [17]. We performed 10 random splits of each dataset into two equal parts and performed CluBCpG analysis on B cell vs. B cell, monocyte vs. monocyte, and B cell vs. monocyte (Fig. 2a). Relative to analyses comparing a single cell type to itself, CluBCpG identified > 20-fold more sample-specific read clusters when comparing different cell types (*P* = 6 × 10^−52^, one-way ANOVA with Tukey post hoc test) (Fig. 2b). We performed an analogous analysis using WGBS data from human neurons and glia [18] (15X genome-wide coverage) and found a similar result (*P* = 2.2 × 10^−31^) (Fig. 2c). Importantly, the B cells and monocytes were isolated from one individual (a 37-year-old male) (Supplementary Table 1), and the neuron and glia samples were likewise isolated from one individual (a 40-year-old male), ruling out potential confounding by age, sex, etc. These data therefore support the hypothesis that, rather than arising by chance or reflecting stochastic processes, most sample-specific clusters identified by CluBCpG are associated with cell type.

To assess the frequency of cell type-specific signals throughout the genome, we calculated the number of clusters containing B cell-specific reads, monocyte-specific reads, or both in the full B cell and monocyte datasets; the vast majority of clusters are common to both cell types (Supplementary Figure 2a). Genome-wide, across 4.1M informative bins, CluBCpG detected an average of 1.7 clusters per bin (Supplementary Figure 2b). Only 8% of bins contained a B cell- and/or monocyte-specific cluster, and these were generally exclusive; only 5% of bins with a cell type-specific cluster had clusters specific to both cell types (Fig. 2d). Despite its lower sequencing depth (15X), the neuron vs. glia data set yielded similar results, with 9% of covered bins containing neuron- and/or glia-specific clusters, and 4% of these containing both (Fig. 2e). Interestingly, comparing read cluster patterns unique to a cell type vs. shared by both cell types showed that shared patterns tend to be fully methylated, whereas cell type-specific patterns are broadly biased towards other states (Supplementary Figure 3).

One might suppose that many of the regions detected by CluBCpG correspond to conventional cell type-specific differentially methylated regions (DMRs). To test this, we identified B cell vs. monocyte DMRs using Dispersion Shrinkage for Sequencing data (DSS) [19, 20], a state-of-the-art, statistically rigorous software package to identify DMRs in WGBS data without the requirement of sample replicates. Using DSS thresholds of a minimum DMR length ≥ 50 bp and ≥ 2 CpGs per DMR (*P* < 10^−8^), we find that < 10% of bins with B cell- or monocyte-specific clusters overlap DMRs (Fig. 2f). Even after applying a greatly relaxed *P* value significance threshold within DSS (*P* < 0.05), 70% of bins identified by CluBCpG are not found in DMRs (Supplementary Figure 4a-c). To test whether cell type-specific clusters might simply correspond to very short DMRs, we ran DSS using various minimum DMR length requirements and found a similarly low overlap of clusters with DMRs (Supplementary Figure 4d). Together, these results indicate that genomic regions exhibiting cell type-specific read patterns are largely distinct from DMRs. Indeed, relative to gene regions, the distribution of bins with cell type-specific clusters is significantly different from that of DMRs (*P* < 7.9 × 10^−252^–6.8 × 10^−42^, *χ*^2^ test) (Supplementary Figure 4e-f).

Given the enrichment of cell type-specific bins in intergenic regions (Supplementary Figure 4f), we hypothesized that these may correspond to enhancers. We compared the number of bins with a cell type-specific cluster overlapping active enhancer regions [21] to a random, background set of bins and found that in both the brain (neuron/glia) and blood (B cell/monocyte) datasets the bins are indeed enriched at corresponding cell type-specific active enhancers (Supplementary Figure 4g; all bins; *P* < 1 × 10^−300^). These enrichments remain after excluding bins overlapping a DMR (Supplementary Figure 4g; non-DMR bins; *P* = 1.6 × 10^−157^ for neuron/glia and *P* = 5.1 × 10^−266^ for B cell/monocytes). We further tested the cell type specificity of these enrichments, finding that brain enhancers overlap more with the neuron/glia clusters, while blood enhancers overlap more with the B cell/monocyte clusters (Supplementary Figure 4h).

For all cell type-specific clusters, we assessed the proportional representation of sample reads per clusters (i.e., within each bin, the number of sample-specific reads in a cluster divided by all reads from that sample) (Fig. 2g). Most sample-specific clusters comprise a minority (< 50%) of the reads from that sample (which we call minor clusters). Major clusters, comprising a majority (≥ 50%) of reads from that sample, are relatively rare. One interpretation is that cell type-specific major clusters may reflect genomic regions that distinguish the two cell types in a comparison, whereas minor clusters may reflect cellular sub-types (different B cell sub-types for example). In support of this, we found that the enrichment of cluster bins with enhancers is almost entirely driven by major clusters, in both tissues (Supplementary Figure 4g; *P* < 1 × 10^−300^).

To test whether bins with cell type-specific clusters play a regulatory role, we utilized the Genomic Regions Enrichment of Annotations Tool (GREAT) [22] to perform gene ontology (GO) analysis on bins containing sample-specific clusters. Several of the most statistically significant biological process terms were common to bins containing B cell- and monocyte-specific clusters and were consistent with these cell type’s shared roles in immune function (Fig. 2h). But each was also enriched for terms that associate specifically with their cellular identity, such as B cell receptor signaling in B cells and leukocyte degranulation in monocytes. Top GO process terms associated with genomic bins containing neuron- and glia-specific clusters showed no overlap and were likewise associated with their cell type, including retrograde transport in neurons and granule cell precursor proliferation in glia (Fig. 2i).

We repeated the B cell vs. monocyte GO analysis including only the 25,429 and 22,932 bins containing B cell- or monocyte-specific major clusters, respectively (Fig. 2j). Compared to the analysis including all bins with unique clusters (Fig. 2h), more top process terms were unique to one of the two cell types and all were directly related to B cell or monocyte functions, consistent with our interpretation that major clusters correspond to regions that best discriminate two cell types in a comparison.

Together, these data support the hypothesis that, within bulk WGBS data, read-level CpG methylation patterns identified by CluBCpG are indicative of different cell types. Of note, whereas conventional analysis of WGBS data is largely based on calculation of average methylation levels at specific CpG sites or regions, the above analyses are based solely on differences in read-level methylation *patterns*, without regard to average methylation levels of clusters or bins.

### Accurate imputation of CpG methylation values on individual WGBS reads

**Fig. 3:**
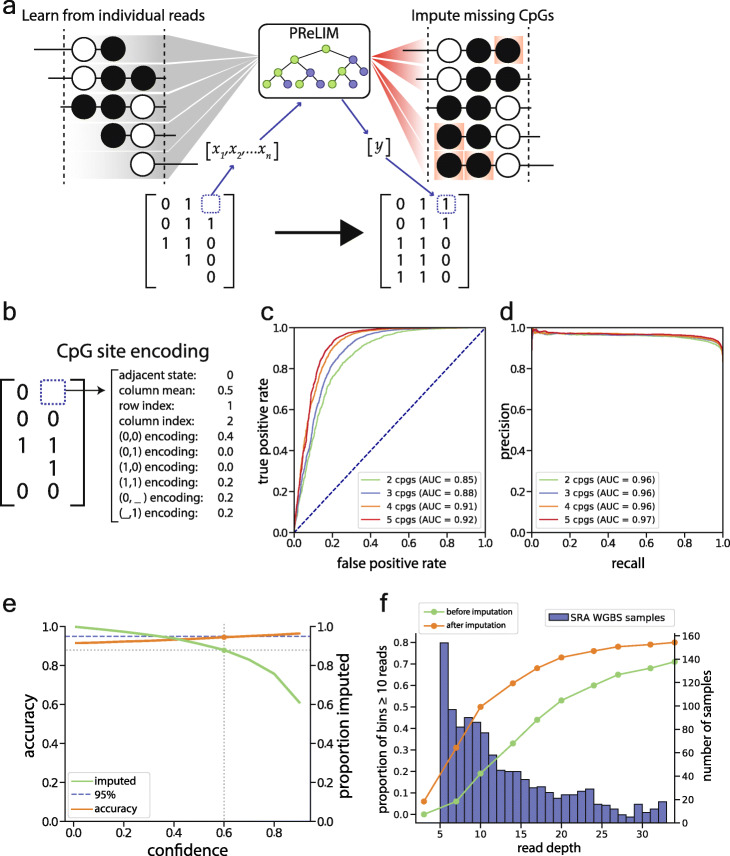
Precise Read-level Imputation of Methylation (PReLIM) imputes missing methylation values at the read level. **a** Conceptual illustration of PReLIM. During training, PReLIM learns about associations of CpG methylation patterns within and among millions of reads from a given dataset. PReLIM then uses this knowledge to impute missing CpG values for all reads overlapping each 100-bp bin, enabling the generation of complete matrices that can be used by CluBCpG. **b** PReLIM expands each individual CpG site to a 1D vector which contains all the information for that CpG site in the context of all other reads in that bin. Read encodings are the relative proportions of each possible type of methylation pattern found in the matrix. **c** Receiver operating characteristic plot showing PReLIM’s performance on the 20% of mouse neuron data held out during training. **d** Corresponding precision-recall plot. **e** Trade-off plot illustrating associations between prediction confidence, prediction accuracy, and proportion of imputations achieved. Dotted lines show that, for this data set, considering only predictions with confidence > 0.6 enables 90% of missing values to be imputed at 95% accuracy. **f** Line plots (scale on left axis) show that imputation by PReLIM enables substantial gains in the proportion of genomic bins meeting CluBCpG coverage requirements on the ENCODE B cell data. Bar plot (scale on right axis) shows estimated coverage level of WGBS libraries currently deposited in the NCBI SRA; libraries with less than 5X coverage are not shown. For the majority of these datasets, PReLIM can increase coverage by 50–100%

A limitation of CluBCpG is that, due to the random nature of shotgun sequencing, many WGBS reads do not cover all CpG sites within a bin and are not fully utilized. Since the number of informative bins decreases dramatically with lower genome-wide sequencing depth (Supplementary Figure 5), identifying cell type-specific signals in WGBS data requires high sequencing depth. To make CluBCpG amenable to a wide range of existing WGBS datasets, we wished to impute methylation values at “missing” CpGs on reads partially overlapping each 100-bp bin. Machine learning tools have been developed to predict DNA methylation at un-assayed CpG sites in WGBS experiments, but these predict a methylation value at the sample library level, not on individual reads [23–26]. We set out to create a model which could learn from the millions of reads within each WGBS data set and impute “missing” CpG methylation values at the read level (Fig. 3a).

The result is a software package called Precise Read-Level Imputation of Methylation (PReLIM), which implements a random forest machine learning model. PReLIM was initially developed using publicly available WGBS data from mouse neurons and glia [27] (see the “Methods” section for a detailed description). In short, we generated training data for each cell type individually by identifying all bins with at least 10 informative reads. To mimic actual patterns of “missingness,” where the ground truth is known, we used bins containing incomplete reads to generate masks and overlaid these on fully covered bins. Each CpG site was encoded into a 1D vector (Fig. 3b) including data such as the adjacent CpG state, mean methylation of all CpGs at that genomic position, number of CpGs on that read, and the proportions of all CpG patterns within the bin. These encoded data were split and used for 5-fold cross-validation. We tested multiple machine learning algorithms: K-nearest neighbors, logistic regression, random forest, and neural networks. As random forest and neural networks both performed the best and at similar levels (Supplementary Figure 6a-b), we opted to employ random forest as it is less computationally intensive. We additionally tested if PReLIM provides better predictions than simply using the column mean (average methylation at a given CpG site) as the predictor. Indeed, PReLIM’s predictions yielded a significantly higher area under the receiver operating characteristic curve (AUC) (*P* < 0.01) when tested in both neurons and glia (Supplementary Figure 6c).

Across all CpGs, PReLIM delivers an AUC of 0.85 or better, and performance increased with bin CpG density (Fig. 3c); the area under the precision-recall curve (AUPRC) was 0.96 (Fig. 3d). Recognizing that attempting to impute all CpGs compromises overall performance, we opted to achieve higher accuracy by limiting methylation calls to CpG sites with a high prediction confidence *C* = | 2 (*y* − 0.5) |, where *y* is the probability of methylation (from 0 to 1) predicted by the model. Considering only predictions with a confidence of > 0.6 enables imputation of 90% of the missing values, with 95% accuracy (Fig. 3e); this approach was utilized to maintain prediction accuracies at 95% in subsequent analyses.

Without utilizing confidence cut-offs, we asked whether a model trained on one library could be used to impute methylation on WGBS reads from a different library and found that, while possible, performance is generally best when each model is trained on the library of interest (Supplementary Figure 7a). We also tested if models need to be trained in a chromosome-specific fashion but observed similar performances across chromosomes (Supplementary Figure 7b) and therefore opted to train the models genome-wide for each sample.

To determine the gains in coverage attainable by PReLIM, we created a downsampled version of the ENCODE B cell data [17] and calculated the numbers of fully covered bins (i.e., ≥ 10 complete reads) before and after imputation. The biggest proportional gains from imputation are achieved when genome-wide read depth is between 7X (390% gain) and 20X (37% gain) (Fig. 3f, green and orange lines, left axis). Many WGBS datasets in the NCBI sequence repository archive (SRA) fall within this range (Fig. 3f, blue bars, right axis). Similar gains from imputation were observed on the human neuron and glia datasets [18] (Supplementary Figure 8). Together, these results demonstrate that PReLIM can efficiently and accurately impute read-level CpG methylation values, suggesting the potential to recover substantial latent information in existing and future WGBS datasets.

### PReLIM improves power of multiple downstream analyses

To evaluate this capability, we reanalyzed the human neuron and glia datasets [18] after imputation with PReLIM, achieving a 104% increase in the number of bins passing CluBCpG coverage criteria (Supplementary Figure 9a). Even for the ENCODE B cell and monocyte datasets (with their higher initial read depth), CluBCpG coverage increased by 22% (Supplementary Figure 9b). Repeating our GO analysis on the augmented neuron and glia datasets showed that imputation generally increased the statistical significance of the term enrichments (*P* < 2.5 × 10^−67^ and *P* < 1.2 × 10^−36^; Wilcoxon signed rank test) (Supplementary Figure 9c-d), indicating that imputed data generally agrees with and strengthens the results obtained without imputation.

**Fig. 4:**
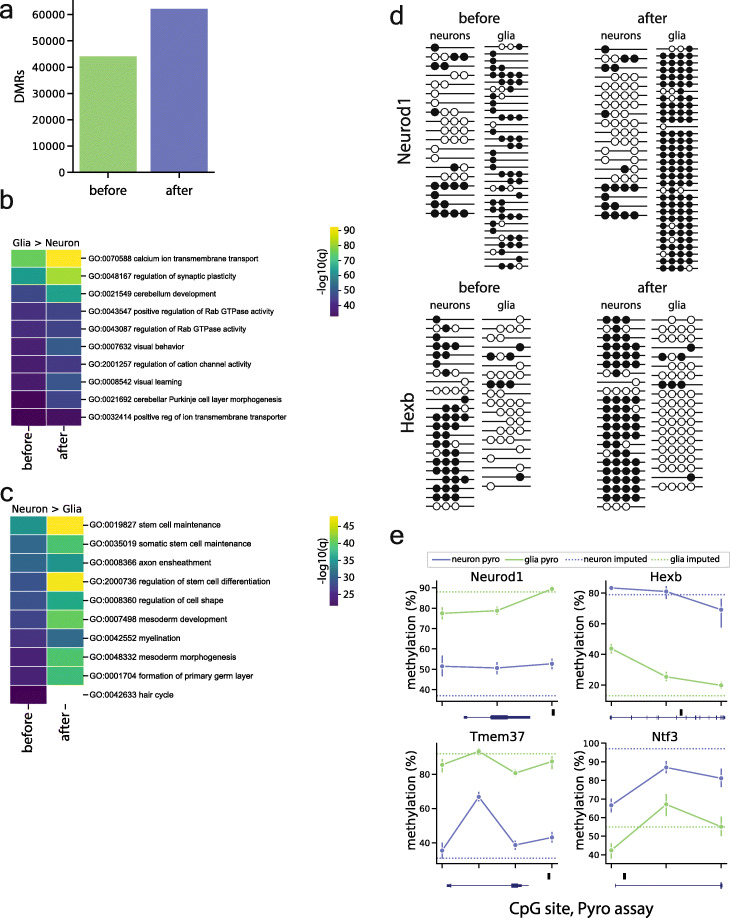
PReLIM increases power and coverage of WGBS datasets. **a** Differentially methylated regions (DMRs) identified in the mouse neuron vs. glia WGBS dataset before and after imputation. **b**, **c** Heatmap showing the top 10 GO biological process terms for DMRs with **b** lower methylation in neurons and **c** lower methylation in glia, before and after imputation by PReLIM; analysis was conducted using GREAT, color represents the -log10 *q* value. PReLIM generally increases the statistical significance of the GO terms. **d** Examples of tanghulu plots showing WGBS reads at DMRs identified only post-imputation; rows and columns represent reads and CpG sites, respectively. Filled and empty circles represent methylated and unmethylated CpGs. **e** Examples of bisulfite pyrosequencing results of DMRs identified only post-imputation. Each point represents a single CpG site in the pyro assay, within the DMR. Horizontal dotted lines indicate average cell type-specific methylation across the DMR, from the WGBS data following PReLIM imputation. DMR positions relative to genes are depicted below each plot. Black box indicates DMR location, blue gene-body schematic is oriented 5′ to 3′

In addition to improving coverage for CluBCpG, we hypothesized that PReLIM might increase the power of conventional WGBS analyses such as identification of DMRs. Using publicly available WGBS data of mouse neuron and glia from Lister et al. [27], we utilized DSS to identify DMRs before and after imputation. Setting a conservative significance threshold within DSS (*P* < 10^−8^), PReLIM enabled the identification of 41% more DMRs (Fig. 4a). As a negative control, we generated multiple splits and evaluated the CpG-specific *P* value distribution from DSS for neuron vs. neuron and glia vs. glia, before and after imputation. The results (Supplementary Figure 10) show that PReLIM does not introduce inflation under the null. We used GREAT to perform GO analysis on the DMRs identified before and after imputation, finding higher statistical significance following imputation (*P* < 1.3 × 10^−4^ and *P* < 4.3 × 10^−4^; Wilcoxon signed rank test) (Fig. 4b, c). To visualize how PReLIM enables identification of more DMRs, we generated tanghulu plots of some regions before and after imputation (Fig. 4d), demonstrating large information gains of both methylated and unmethylated states. To independently validate these newly identified DMRs, we used NeuN immunolabeling and FACS sorting to isolate neuronal and non-neuronal DNA from mouse cortex and evaluated methylation by bisulfite pyrosequencing [28]. Of 10 regions for which pyrosequencing assays passed quality control, 100% validated as DMRs, with correct polarity (Fig. 4e, Supplementary Figure 9e). Overall, these data demonstrate that PReLIM can uncover substantial latent information from WGBS datasets. It not only provides increased coverage for read-level analyses (such as CluBCpG), but also increases the effective read depth of WGBS data, increasing the power of other downstream analyses.

### CpG read clusters precisely estimate proportions of cell mixtures

**Fig. 5:**
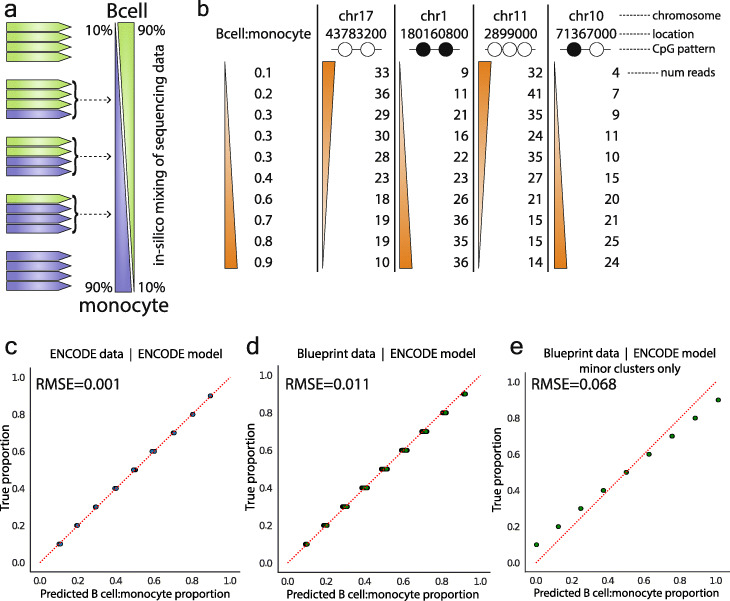
CluBCpG enables proportional estimation of in silico cell mixtures. **a** Illustration of how individual reads from pure B cell and monocyte WGBS libraries were mixed computationally to create synthetic cell mixtures. **b** Examples of data columns from the ENCODE training data used to fit a linear model. B cell to monocyte proportion is the dependent variable. Each column represents a read-level methylation pattern within a bin, and the number of reads showing that pattern in the bin. **c** Predicted B cell to monocyte proportion vs. the true proportion on a subset of 20% ENCODE data held out from training of the linear regression model; note at each position 10 points are overlapping one another. **d** Predicted B cell to monocyte proportion vs. true proportion for all Blueprint B cell and monocyte data. Predictions were based on the linear model fit on the ENCODE data. **e** Predicted B cell to monocyte proportion vs. true proportion for all Blueprint B cell and monocyte data using only minor clusters. For **c**–**e**, the diagonal, red dotted line is the line of identity

If the read patterns being identified by ClubCpG are indeed cell type-specific signatures, we hypothesized that these patterns would enable estimation of cell proportions in mixtures of cells. To test this, we created multiple in silico mixtures of cells by computationally mixing WGBS reads of B cells and monocytes [17] randomly in proportions ranging from 10:90 to 90:10 (Fig. 5a). As in the previous analysis (Fig. 2), these ENCODE data are based on B cells and monocytes isolated from one human subject. We performed analysis with CluBCpG on each mixture and used the identified numbers of reads in each cluster as features in a model to predict the B cell to monocyte proportion (Fig. 5b) (recall that each cluster has a specific CpG methylation pattern and genomic location). To make our analysis orthogonal to DMRs, we excluded all features from bins overlapping a DMR. We performed dimensionality reduction using principal component analysis (PCA) and kept the top 20 components for use in a multivariate linear regression model (Supplementary Figure 11a-b). To verify our model is not overfitting, we carried out 5-fold cross-validation, consistently obtaining a root mean squared error (RMSE) of 0.001 (Fig. 5c) on each testing set which was held back from training. We then assessed the external validity of this model based on ENCODE data, testing if it could accurately predict cellular composition in an independent B cell and monocyte WGBS dataset from the Blueprint Epigenome project [29] (different donors; WGBS performed by a different lab) (Supplementary Table 1). Using the linear regression model trained on ENCODE data to predict the proportions in the Blueprint data achieved near-perfect accuracy (RMSE = 0.011) (Fig. 5d, Supplementary Figure 11c). Note that the ENCODE B cell and monocyte data are from one (37-year-old male) individual, and the Blueprint data are from at least one female (65–70 years old) (Supplementary Table 1); hence, the agreement of these models cannot be explained by confounders such as age or sex. We next tested if minor clusters (Fig. 2g) carry cell type-specific information, fitting another linear model using only minor cluster information, not overlapping a DMR, from the Encode data. Testing it against the Blueprint data, the model performed with a RMSE of 0.068 (Fig. 5e), indicating that, although a subset may reflect stochastic variants, some minor clusters do contain cell type-specific information. As negative controls, we generated random data and permuted the proportion labels of the Blueprint data. In both cases, testing on the ENCODE-trained model showed no predictive relationship (RMSE = 0.31 and 0.37, Supplementary Figure 11d-f). These results provide compelling evidence that, independent of cell type DMRs, read clusters identified by CluBCpG represent cell type-specific signatures within WGBS data.

### CpG read clusters improve prediction of gene expression

**Fig. 6:**
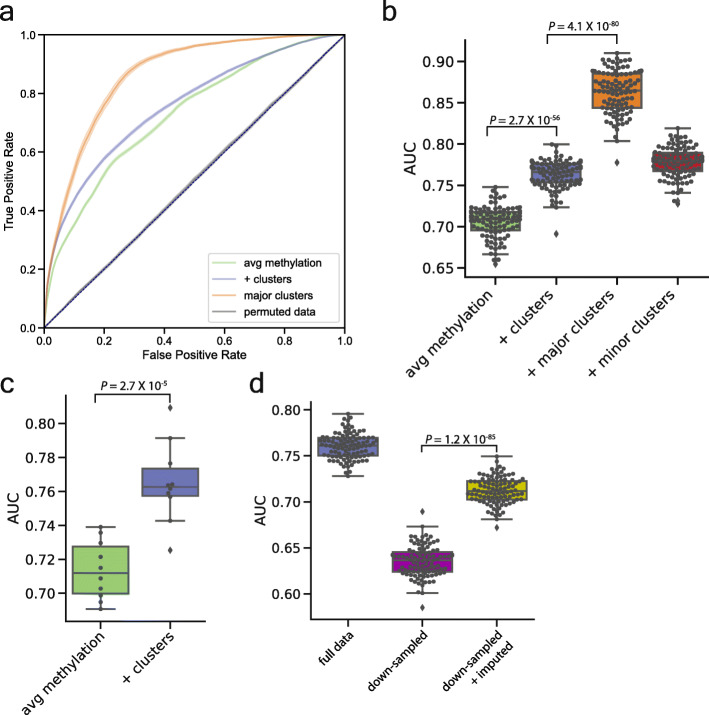
CluBCpG read clusters improve prediction of gene expression. **a** Receiver operating characteristic (ROC) curves of a random forest model trained on promoter average methylation alone (green line), promoter average methylation plus cluster information (purple line), promoter average methylation plus cluster information on the subset of gene promoters containing a major cluster (orange line), and promoter average methylation plus cluster information in which the class labels were permuted (gray line). Shading represents the 95% confidence interval of 100 random train-test splits. **b**–**d** Box and whisker plot overlaid with individual points showing the area under the ROC curve for train-test splits. Whiskers extend to 1.5x the intra-quartile range. **c** AUC results from a 10-fold nested cross-validation strategy that was used to ensure the models were not overfitting. **d** Downsampled data were the full B cell vs. monocyte dataset randomly reduced to 9X genome-wide coverage. Statistical tests: *t* test, two-tailed

To directly test their role in epigenetic regulation, we asked whether the read clusters inferred by CluBCpG could be used to predict gene expression. Gene expression data were obtained for the ENCODE B cell and monocyte samples [17]. We focused our analysis on the promoters of 3750 genes with differential expression. Our analysis builds upon a previously reported approach for predicting expression differences in a binary fashion (i.e., predicting which cell type has higher expression of a given gene) using a random forest model [30]. For each gene, we constructed a set of features detailing the identified clusters around its promoter (Supplementary Table 2). For a baseline comparison, the initial model was based on average promoter methylation, without considering clusters. Promoter methylation alone achieved a mean AUC of 0.69 (Fig. 6a, b, green). Including cluster information increased the mean AUC to 0.75 (Fig. 6a, b, purple; *P* = 2.7 × 10^−56^). As a negative control, we permuted the class label of our full training data set; as expected, no predictive relationship was observed (AUC = 0.5, Fig. 6a, dark gray). To further verify that the models are not overfitting the data, we implemented a 10-fold nested cross-validation procedure in which models are independently optimized for each fold of training data [31]. The results (Fig. 6c) mirrored the original analysis (Fig. 6b), corroborating that cluster information increases predictive performance.

Based on studies evaluating associations between regional methylation and gene expression [30], our expression analyses utilized a promoter window of ± 3 kb. However, to ensure this promoter window is not too large (potentially diluting the functional methylation differences), we compared our baseline model (transcription start site (TSS) ± 3 kb) against a model trained using average methylation levels in a window encompassing 500 bp upstream of the TSS. The baseline model using the larger promoter window possessed more predictive power (mean AUC 0.70 vs. 0.63; *P* = 5.8 × 10^−58^) (Supplemental Figure 12a-b). This finding is consistent with other studies [30] and suggests that the ability of cluster information to improve expression predictions is not dependent on the choice of promoter window.

We wished to test whether the improved ability to predict expression is inherent to the methylation patterns within the clusters, or simply reflects the inclusion of CpG-level data. We therefore calculated the frequencies of CpG methylation-deciles for each gene promoter and included these in the baseline random forests model (Supplementary Table 3). Surprisingly, the inclusion of these data *decreased* the performance of the model (AUC 0.65 vs. 0.73) (Supplementary Figure 12c), indicating that the cluster pattern information does indeed contain cell type-specific information beyond that in the individual CpG methylation levels.

Given our hypothesis that major clusters (Fig. 2g) best distinguish predominant cell types in a sample, we predicted that bins with major clusters would yield better gene expression predictions. Indeed, evaluating only the 716 gene promoters that include a major cluster improved model accuracy to a median AUC of 0.86 (Fig. 6a, b, orange; *P* = 4.1 × 10^−80^ vs. all clusters). Conversely, no improvement was found when evaluating the gene promoters containing only minor clusters (Fig. 6b, red).

Lastly, we tested whether applying PReLIM to low-coverage datasets can improve cluster-based prediction of gene expression. We downsampled the ENCODE dataset to 9X average read depth and performed CluBCpG analysis before and after imputation. Remarkably, the reduced performance of the downsampled data set was partially rescued following imputation by PReLIM (*P* = 1.2 × 10^−85^) (Fig. 6d). In addition to providing further evidence that PReLIM improves the ability to draw biological inferences from WGBS data, these analyses indicate that the cell type-specific methylation patterns identified by CluBCpG play a role in regulating cell type-specific gene expression, above and beyond that which can be predicted by promoter methylation alone.

## Discussion

Current understanding of mammalian developmental epigenetics holds that, following lineage-specific transcriptional activation, de novo DNA methylation occurs as a long-term silencing mechanism, resulting in DMRs within gene regulatory elements [1, 32]. Here we demonstrate that, in WGBS data on synthetic mixtures of isolated cell types, read-level patterns of CpG methylation, which are largely distinct from DMRs, associate with cell type. Our findings align with previous studies indicating that methylation at individual CpG sites can regulate gene expression [33–36], and suggest that regulation of gene expression by CpG methylation is more complex than has been generally appreciated. For example, it is possible that in addition to transcriptional silencing at DMRs, single CpG dinucleotides could be targeted for methylation, blocking (or enabling) recognition by specific DNA binding proteins in a lineage-specific fashion.

One remarkable finding from our study is that many of the genomic bins containing cell type-specific clusters do not overlap cell type-specific DMRs. However, given that DMRs are first identified by performing a statistical test at each CpG site [19], it is understandable that read-level heterogeneity causes high variance, compromising statistical significance. Since DMR finding methods were not developed to detect this type of in-library heterogeneity, CluBCpG complements traditional DMR analyses.

Previous reports have analyzed individual reads from WGBS or RRBS data to develop more fine-grained analytical approaches that go beyond locally averaged methylation [5, 10]. These, however, all build upon the conjecture that read-level methylation variation largely reflects stochastic processes [37]. Disorder is implicit, for example, in the term “methylation entropy.” In contrast, our data provide support for the hypothesis that intermolecular variations in CpG methylation patterns carry information, are established during differentiation, and reflect heterogeneity of cell types (and sub-types). Some of the variation detected as minor clusters in our CluBCpG analysis could reflect stochastic processes. However, in multiple instances, our findings demonstrate that some minor clusters contain cell type-specific information. We show that using only minor cluster bins still enables accurate estimation of cellular proportions in synthetic mixtures (Fig. 5e). Minor clusters also enable improved prediction of gene expression, beyond promoter methylation alone (Fig. 6b). While our analyses do not rule out that some of this variation arises stochastically, they do demonstrate that the heterogeneity of site-specific CpG methylation patterns in WGBS data is strongly associated with cell type, suggesting a previously unrecognized level of complexity in epigenetic regulation by CpG methylation.

In addition to enabling such novel theoretical insights, CluBCpG has many potential practical applications. An early version of this approach was successfully utilized to infer cell type-specific methylation differences among mouse hypothalamic neurons, a subset of which were affected by cell type-specific knockout of *Dnmt3a* [38]. With sufficient high-quality WGBS data to construct cell type-specific references, CluBCpG could be utilized to develop a new state-of-the-art, highly accurate cellular deconvolution approach. Additionally, CluBCpG patterns from consecutive bins could potentially be linked together to construct and identify methylation haplotypes present over genomic long genomic distances [12]. Such future applications, however, are beyond the scope of this report.

Our analyses also demonstrate the remarkable ability of our new software tool, PReLIM, to impute unknown CpG methylation values at the read level. Previous methods to impute CpG methylation have focused on predicting at the sample level [23, 26, 39]. To our knowledge, PReLIM is the first machine learning tool to impute unknown CpG states on individual WGBS reads. Because it enables construction of “complete” matrices for all reads within each genomic bin, PReLIM will enable future computational innovations employing matrix operations on WGBS data. Our extensive work developing this tool provided a few “key insights” that enable PReLIM to deliver outstanding prediction accuracy: (1) training occurs on a random sample of the entire genome (separately for each CpG density) providing the classifier access to extensive information about heterogeneity of methylation patterns across a wide range of genomic contexts; (2) by automatically optimizing model hyperparameters, PReLIM requires no user-input during training; (3) by restricting predictions to only high-confidence methylation calls, PReLIM enables excellent accuracy while still imputing the vast majority of missing CpG methylation values. We are providing CluBCpG and PReLIM as open-source tools to the scientific community and anticipate that they will effectively complement existing and future WGBS analytical approaches.

## Methods

### CluBCpG and PReLIM availability

The source code (along with installation and usage instructions) is available on the Waterland lab GitHub repo at the following URLs: https://github.com/waterlandlab/CluBCpG and https://github.com/waterlandlab/PReLIM. Full documentation and user guides are also available at https://clubcpg.readthedocs.io/ and https://PReLIM.readthedocs.io/.

### WGBS preprocessing and analysis pipeline

A standard WGBS data analysis pipeline was followed. First, raw fastq files were quality trimmed using TrimGalore (v0.4.3) with default settings. Reads were aligned to the full genome (mm10 for mouse and hg38 for human) using Bismark (v0.18.1) [15]. Mapped BAM files were then query name sorted and deduplicated using Picard (v0.2.10.10) and finally coordinate sorted using samtools (v1.9) [40].

### CluBCpG analytical approach

CluBCpG runs its analysis in two phases. Phase 1 calculates the number of reads fully covering each bin. The output from phase 1 is then used in phase 2 where reads are extracted from each bin and clustered together. Phase 1 allows the acceleration of phase 2. Both phases of analysis can be parallelized across multiple CPUs and/or CPU cores.

#### Phase 1

Each chromosome is divided into 100-bp non-overlapping bins. For each bin, all reads are extracted, and the number of informative reads is calculated and reported. CluBCpG considers only reads mapping onto the reference genome without indels, e.g., either perfect matches or allowing for mismatches within the default parameters for Bismark. This phase makes no assumption about the chromosome sizes or number of chromosomes, allowing it to be performed on WGBS data from any species. The output from phase 1 can be filtered for a desired coverage level or CpG density. This output is then used as an input for phase 2.

#### Phase 2

Reads from each bin are extracted from one or two bam files. If two bam files are provided, sufficient read coverage is verified from the second bam file. If read coverage is met, reads are combined into one matrix and their sample of origin is tracked. The DBSCAN method from scikit-learn [41] is used to rapidly cluster reads by identity. Identified clusters and corresponding data are all saved to an output file.

A detailed user guide along with usage examples is available at https://clubcpg.readthedocs.io/.

### Splitting samples and performing sample-type comparisons

Samtools was used to split reads from each bam file into two different fractions, each containing 50% of the mapped reads. Each random split was seeded with a different integer starting at one and incrementing by one each time. Bin coverage was calculated using CluBCpG’s “clubcpg-coverage” tool, and this output was filtered for bins containing ≥ 10 reads and ≥ 2 CpGs. CluBCpG’s “clubcpg-cluster” tool was then used to perform read analysis between each pair of sampled BAM files.

### GREAT analysis

Bins of interest were exported as a BED file. UCSC’s “liftOver” command-line tool was used to convert the hg38 coordinates in the BED files to hg19 coordinates. These converted BED files were uploaded to GREAT (v3.0.0) [22] for analysis. GREAT was run on its default settings. Due to a large number of regions being analyzed, the “region-based binomial view” was used, as suggested by the developers. Significant GO Biological Process terms were exported.

### DSS analysis

Bismark methylation extraction was used to generate coverage files from the Bismark-aligned BAM files. The coverage files were utilized to prepare a DSS input file containing four columns as instructed by the DSS user guide: chromosome, CpG position, total number of reads, and number of methylated reads. DSS [19] was run using a minimum DMR length of 50 bp and a minimum of 2 CpGs per DMR. Unless otherwise noted, we applied a *P* value of < 10^−8^ as a threshold for significance within the DSS software package.

### PReLIM training and validation

#### Data collection

We used WGBS data from Lister et al. [27] from mouse neuron and glia. WGBS reads were prepared in the aforementioned fashion, resulting in coordinated sorted BAM files. We then partitioned the genome into 100-bp bins. Segments of reads overlapping the CpG loci in a bin were placed into that bin. We represented the reads overlapping each bin using a matrix, where columns are CpG loci and rows are individual reads. We represented a methylated CpG with a 1 and an unmethylated CpG with a 0. If a read did not cover a CpG in the bin, we represented the missing CpG with a − 1. In this way, we obtained a list of CpG matrices. To train PReLIM to predict the methylation status of missing individual CpGs at the read level, we needed a way to know the true methylation status of a CpG. We achieved this by first creating a set of “masks” to artificially hide data. In order to create a set of masks that were representative of the data, we looked at all the CpG matrices in the data and recorded the positions of the missing CpGs in each matrix. We only recorded masks that had at least 1 missing CpG. We then needed a set of matrices that had no missing values in them that we could apply our masks to. To collect these complete matrices, we looked at all the matrices in the data and removed any reads with missing values in them. For each of these complete matrices, we found a mask that had the same dimensions. We then used our masks to artificially cover up values in the complete matrices, and for each CpG that was covered up, we recorded its known methylation state. For each artificially missing CpG in a matrix, we used the artificially masked matrix and recorded the mean methylation in the corresponding row and column, the current column and row index of the CpG, the methylation states of every CpG in the current row, and the “read encodings,” which are the relative proportions of each type of methylation pattern found in the matrix (see example in Fig. 3b). These values constituted the feature space data corresponding to each of the missing CpGs, serving to capture as much local methylation information as possible while still allowing the same model to be used on bins with different read coverages.

#### PReLIM training and testing

PReLIM uses scikit-learn’s [41] random forest implementation. We performed a grid search to find the optimal hyperparameters for the model (number of trees [10, 50, 100, 500, 1000], maximum depth of trees [1, 5, 10, 20, 30]). We split the data into two partitions: training data (80%) and validation data (20%). Scikit-learn’s standard 5-fold validation was used for each hyperparameter setting. We created a model for the most common CpG densities, that is, 100-bp bins with 2, 3, 4, and 5 CpGs.

#### Comparison to Naïve Bayes model

We trained both PReLIM and a Gaussian Naive Bayes (NB) model on mouse neuron and glia datasets. For all of these datasets, we considered only bins with 2 CpGs. For all models, 10,000 data points were included in the data set. The NB model was trained only on the observed mean of each CpG site, while PReLIM was trained on all features. The NB model thus tries to predict the methylation of an individual CpG site at the read level given the observed mean of the other CpGs at this site. The prior for the NB model is learned from the training data. For each data set, we used 5-fold cross-validation to evaluate the performance of both models. We used the area under the receiver operating characteristic curve as a metric to compare the performance of the models.

#### Accuracy evaluation

PReLIM emits the estimated probability that a given CpG is methylated. A probability close to 1 means that PReLIM is more confident that the CpG is methylated, and a probability closer to 0 indicates that PReLIM is more confident that the CpG is unmethylated. A probability close to 0.5 means that PReLIM is unsure. We evaluated PReLIM’s accuracy performance as a function of confidence. We defined “confidence” as the absolute value of twice the distance of the probability to 0.5, i.e., *C* = | 2 (*y* − 0.5) |, where *y* is the estimated probability of methylation.

#### Imputation

To impute missing data from a trained PReLIM model, PReLIM collects the observed features of a missing CpG site in a matrix, feeds these features through its model, and returns the estimated probability of methylation.

#### Testing for DMR inflation under the null following PReLIM

To test whether PReLIM might create inflation of the DSS test statistic under the null, we used the same neuron and glia datasets from before [27] and, for each tissue type, split the reads randomly into two files and applied PReLIM to both. DSS was used to compare the two downsampled files (self vs. self) both before and after imputation; ten such random splits were evaluated for each tissue type. After imputation, a coverage file was created that had the same format as described under the “DSS analysis” section. Since DSS does not report the *P* value of DMRs, we examined the *P* values used by DSS to call differentially methylated loci (DML). DSS was used as previously described, except for changing the significance threshold to *P* < 1 to capture the full range of CpG-level *P* values.

### Pyrosequencing

Quantitative analysis of novel DMRs identified post-imputation was performed using bisulfite pyrosequencing as previously described [16]. Prior to use, all assays were quantitatively validated against a set of mouse genomic DNA methylation standards. Assay primers and QC data are included in Supplementary Table 4.

### Proportion estimation of in silico cell mixtures

#### Feature collection

Independent B cell and monocyte datasets were obtained from ENCODE [17] and Blueprint [29], both at approximately 30X average sequencing depth. Samtools was used to randomly sample varying proportions of reads from B cell and monocytes and merge them to create an in silico mixture of cells while keeping the same overall average sequencing depth of 30X. Each proportion was created 10–15 times, each time being initialized with a different random seed. Each one of these synthetic mixtures was analyzed using CluBCpG. A total of 7 million read clusters were identified genome-wide. Using the ENCODE B cell and monocyte data, a training set was constructed with each row representing one synthetic mixture and each column (a feature) an observed CpG pattern in a specific 100-bp bin. The value was the number of reads present with that pattern. When constructing the Blueprint data, any feature not found in ENCODE was dropped, and any ENCODE feature not found in Blueprint was added and set to 0. In total, 5.2 million features existed after adjustments. Additionally, approximately 115,000 features present bins that were also identified in DMRs were removed. Principal component analysis (PCA) was performed using scikit-learn with “n_components” = 20.

#### Training and testing for the ENCODE data

Using scikit-learn, 5-fold cross-validation was performed using a linear regression model. The linear model was initialized using scikit-learn default parameters. After cross-validation, a final model was fit on the full ENCODE dataset.

#### Validation on Blueprint data

PCA was applied to the Blueprint data keeping the top 20 components. The model fit to the full ENCODE data was then used to predict the Blueprint proportions. The root mean square error (RMSE) value was calculated by taking the squared root of the output from scikit-learn’s “mean_squared_error” function on the predicted vs. true Blueprint proportions.

### Random forest prediction of gene expression

Fully processed B cell and monocyte expression data were obtained from ENCODE [17]. We selected a set of differentially expressed genes by calculating the Δlog2FPKM by log2 normalizing the FPKM values and subtracting monocytes from B cells. These values were fit to a normal distribution, and genes with a Δlog2FPKM ± 2 standard deviations from the mean were selected. We focused our analysis on gene promoters (transcription start site ± 3 kb) in which we identified cell type-specific clusters. The mean promoter methylation value was calculated by averaging the CpG methylation values for all CpGs within the promoter window. Using CluBCpG results, we created features for each gene, which included the mean promoter methylation value and one column for each CpG pattern observed and its cell type specificity (Supplementary Table 2). Scikit-learn’s RandomForestClassifer was used to build and test each model. The hyperparameters of every random forest model utilized in our study were each tuned independently by performing a grid search along with 10-fold cross-validation testing combinations of various parameters, i.e., numbers of decision trees (n_estimators, 1–100), the function to evaluate the quality of a split (criterion, “gini” and “entropy”), and the maximum depth of a tree (max_depth, 1–100 or none) were evaluated. The optimal parameters for each model were then utilized in the 100 random train-test splits: 80% training and 20% testing. For the nested cross-validation [31], a grid search was utilized on each fold to identify the best performing model. The AUC for each test-fold was then calculated using the optimal model parameters from that fold.

### CpG promoter methylation-decile analysis

Methylation levels of individual CpG sites present within a gene promoter were extracted from the coverage files produced by Bismark. We next quantified the number of CpG sites within each promoter which fell into decile-methylation ranges (0–9.9%, 10–19.9%, etc.) for both B cells and monocytes. The total number of CpGs for each category was then divided by the total number of CpGs within each promoter to produce a normalized proportion of the levels of CpG methylation in each promoter. These data for each gene promoter were then added as additional features to the random forest model data which contained only the average promoter methylation level at each gene promoter.

## Supplementary information

**Additional file 1: Supplementary Table 1.**

**Additional file 2: Supplementary Table 2.**

**Additional file 3: Supplementary Table 3.**

**Additional file 4: Supplementary Table 4.**

**Additional file 5: Supplementary Figures**. **Supplementary Figure 1.** Comparison of existing read-based metrics. Schematic depicting a genomic region containing two CpG sites with different patterns of methylation, but same average methylation, in two different samples. Calculated methylation haplotype load, methylation entropy, and epi-polymorphism metrics are shown to compare how they differentiate (or fail to differentiate) the two samples. **Supplementary Figure 2.** Genome-wide calculation of sample-specific CluBCpG clusters. (a) Bar chart showing total numbers of clusters identified genome wide between ENCODE B cells and monocytes. Bars show total number of clusters found in both samples, B cell only, and monocyte only. (b) Histogram depicting the distribution of the number of clusters per bin from ENCODE B cells and monocytes; x-axis truncated at 10 for clarity. **Supplementary Figure 3.** Predominant CpG patterns differ between shared and cell type-specific clusters. Bar plots showing the total counts of different CpG patterns identified across the full genome. Shared clusters (left) were clusters with patterns found in both B cells and monocytes. Unique clusters (right) were found only in one cell type. Plots are separated by CpG density (i.e. 2, 3, or 4 CpGs/bin). On the y-axes a number 1 indicates a methylated CpG site, 0 is unmethylated. **Supplementary Figure 4.** CluBCpG identified regions are predominantly found outside of DMRs. (a) Venn diagram showing the overlap of CluBCpG-identified bins with read clusters and DSS-identified DMRs using different p-value thresholds. Size of circles scale with number of regions. (b-c) Bar plots showing the ratio of overlapping regions in (a) to (b) total bins with B cell or monocyte specific read clusters and (c) the ratio of overlapping regions to total DMRs. (d) Venn diagrams depicting the overlap between DMRs and bins when adjusting the minimum length threshold within DSS. (e) Bar plot showing the percentage of bins with a cell type-specific cluster (green) and DMRs (purple) overlapping annotated genic features. (f) Bar plot of the odds ratio calculated from the overlaps in (d); annotated genomic features are defined as: promoter = transcription start site (TSS) +/− 3 kb; intragenic = TSS- transcription end site (TES); 3′ = TES +/− 3 kb; intergenic = all other genomic regions. (g) The odds ratio of the overlap between cell type-specific bins and enhancer regions. Non-DMR bins have had all bins overlapping a DMR removed from the analysis. (h) Odds ratio of the overlap between cell type-specific clusters and cell type-specific active enhancers. **Supplementary Figure 5.** CluBCpG informative bins as a function of read coverage. Proportion of bins with ≥10 fully covered reads vs. average read depth of the sequencing data. Calculations were performed on chromosome 19 from ENCODE B cells. **Supplementary Figure 6.** Comparison of multiple machine learning algorithms. Box and whisker plots showing the area under the receiver operating characteristic curve (AUC) for imputation by multiple machine learning algorithms; KNN=K nearest neighbors, LR = logistic regression, NN = neural network, RF = random forest, NB=Naive Bayes. AUCs were calculated from 5-fold cross validation of each model on data from mouse neurons (a) and glia (b). (c) PReLIM was compared against a Naive Bayes model which uses only the Column mean (average methylation at each CpG site) as a feature. No confidence filtering was performed for these comparisons. **Supplementary Figure 7.** PReLIM cross-tissue and cross-chromosome performance. (a) Heatmap showing how a PReLIM model trained on one library performs when predicting on a different library. (b) Heatmap showing accuracy of PReLIM when trained on one mouse neuron chromosome and predicted on all other chromosomes. **Supplementary Figure 8.** Imputation gains in informative bins vs. average sequencing depth. (a-b) Line graphs showing the proportion of bins with ≥10 reads covering all CpGs before and after imputation with PReLIM on human (a) neurons and (b) glia. Calculations were performed using chromosome 19. **Supplementary Figure 9.** Genome-wide imputation gains in informative bins. (a-b) Bar plots showing the number of bins with ≥10 reads genome-wide when analyzing human neuron and glia (a) and human B cells and monocytes (b) before and after imputation. (c-d) Heatmap showing the top 30 GO biological process terms with bins unique to either neuron (c) or glia (d) before and after imputation. Analysis was performed using GREAT, colors represent -log10 of the q-value. (e) Pyrosequencing plots of novel DMRs found only post-imputation using PReLIM on the mouse neuron and glia WGBS data. Each point represents one CpG site and are connected by a line. Horizontal dotted lines indicate average cell type-specific methylation across the DMR, from the WGBS data following PReLIM imputation. **Supplementary Figure 10.** No effect of imputation on DSS P-value distribution under the null. P value distributions for differentially methylated locus (DML) test statistic from DSS, in self vs. self comparisons before and after imputation by PReLIM. Each histogram shows the mean and standard deviation (error bars) across 10 random 50:50 splits of WGBS reads from mouse neuron or glia [27]. In each panel, the full P value distribution (0–1) is shown on the left and the low range (0–0.1) on the right. (a) Neuron vs. neuron. (b) Neuron vs. neuron following imputation by PReLIM. (c) Glia vs. glia. (d) Glia vs. glia following imputation by PReLIM. **Supplementary Figure 11.** Deconvolution PCA and permutation analyses. (a) Scree plot with bars showing proportion of explained variance provided by each of the 20 principal components (PCs). (b-c) Scatter plot of PC1 vs PC2 of the B cell:monocyte synthetic mixtures from ENCODE (b) and Blueprint (c). Colors represents the proportion of the mixture. (d) Scatter plot of PC1 vs PC2 on randomly generated data. (e-f) The predicted proportion vs true proportion on the randomly generated data (e) and Blueprint data with permuted proportion labels (f). **Supplementary Figure 12.** Evaluating alternate strategies to predict gene expression from promoter methylation. Random forest models using methylation data from promoter windows of +/− 3 kb from the transcription start site (TSS) (green) and 500 bp upstream of the TSS (purple) were compared against each other. Box and whisker plots overlaid with individual points showing the area under the ROC curve (AUC) for 100 random train-test splits (a) and 10-fold nested cross validation (b). To test the effect of including methylation levels at each of the individual CpG sites, the +/− 3 k promoter window was broken down into methylation frequencies in decile blocks at the individual CpG-level and tested using 100 random train-test splits (c). Whiskers extend to 1.5x the intra-quartile range.

**Additional file 6.** Review History.

## Data Availability

All WGBS data utilized in this study were obtained from publicly available sources and can be found using the following accession IDs. ENCODE: GSM1186669 (B cell) [42], GSM1186661 (monocyte) [43]; Blueprint: EGAN00001170522 (B cell) [44], EGAN00001390792 (monocyte) [45]; Lister et al.: GSE47966 (mouse neuron [46] and glia [47]); Rizzardi et al.: GSE96612 (human neuron and glia), samples GSM2536543 (Broadman’s area 9 glia) [48] and GSM2536546 (hippocampal neurons) [49]. Enhancer data for datasets E073 and E062 were obtained from the human roadmap epigenome project [17]. Annotations were filtered for “active enhancer” marks from the 18-state model [50]. The source code along with installation and usage instructions is available on the Waterland lab GitHub repo for both CluBCpG [51] and PReLIM [52]. Publication versions of both packages have been deposited on Zenodo [53, 54].
