# Supplementary material for: Identification of cell type-specific methylation signals in bulk whole genome bisulfite sequencing data
Source: Genome Biol. 2020 Jul 1;21:156. doi: 10.1186/s13059-020-02065-5 (PMC7329512; doi:10.1186/s13059-020-02065-5)
Supplement: Supplementary file 5 — Additional file 5: Supplementary Figures. Supplementary Figure 1. Comparison of existing read-based metrics. Schematic depicting a genomic region containing two CpG sites with different patterns of methylation, but same average methylation, in two different samples. Calculated methylation haplotype load, methylation entropy, and epi-polymorphism metrics are shown to compare how they differentiate (or fail to differentiate) the two samples. Supplementary Figure 2. Genome-wide calculation of sample-specific CluBCpG clusters. (a) Bar chart showing total numbers of clusters identified genome wide between ENCODE B cells and monocytes. Bars show total number of clusters found in both samples, B cell only, and monocyte only. (b) Histogram depicting the distribution of the number of clusters per bin from ENCODE B cells and monocytes; x-axis truncated at 10 for clarity. Supplementary Figure 3. Predominant CpG patterns differ between shared and cell type-specific clusters. Bar plots showing the total counts of different CpG patterns identified across the full genome. Shared clusters (left) were clusters with patterns found in both B cells and monocytes. Unique clusters (right) were found only in one cell type. Plots are separated by CpG density (i.e. 2, 3, or 4 CpGs/bin). On the y-axes a number 1 indicates a methylated CpG site, 0 is unmethylated. Supplementary Figure 4. CluBCpG identified regions are predominantly found outside of DMRs. (a) Venn diagram showing the overlap of CluBCpG-identified bins with read clusters and DSS-identified DMRs using different p-value thresholds. Size of circles scale with number of regions. (b-c) Bar plots showing the ratio of overlapping regions in (a) to (b) total bins with B cell or monocyte specific read clusters and (c) the ratio of overlapping regions to total DMRs. (d) Venn diagrams depicting the overlap between DMRs and bins when adjusting the minimum length threshold within DSS. (e) Bar plot showing the percentage of bins with a cell [file 13059_2020_2065_MOESM5_ESM.pdf]

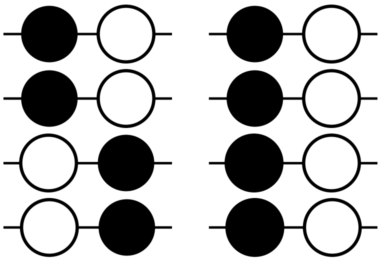

methylation haplotype load	0.17	0.17
methylation entropy	0.5	0
epi-polymorphism	0.5	0
avg methylation	0.5	0.5

Supplementary Figure 1 – Comparison of existing read-based metrics.
Schematic depicting a genomic region containing two CpG sites with different patterns of methylation, but same average methylation, in two different samples. Calculated methylation haplotype load, methylation entropy, and epi-polymorphism metrics are shown to compare how they differentiate (or fail to differentiate) the two samples.

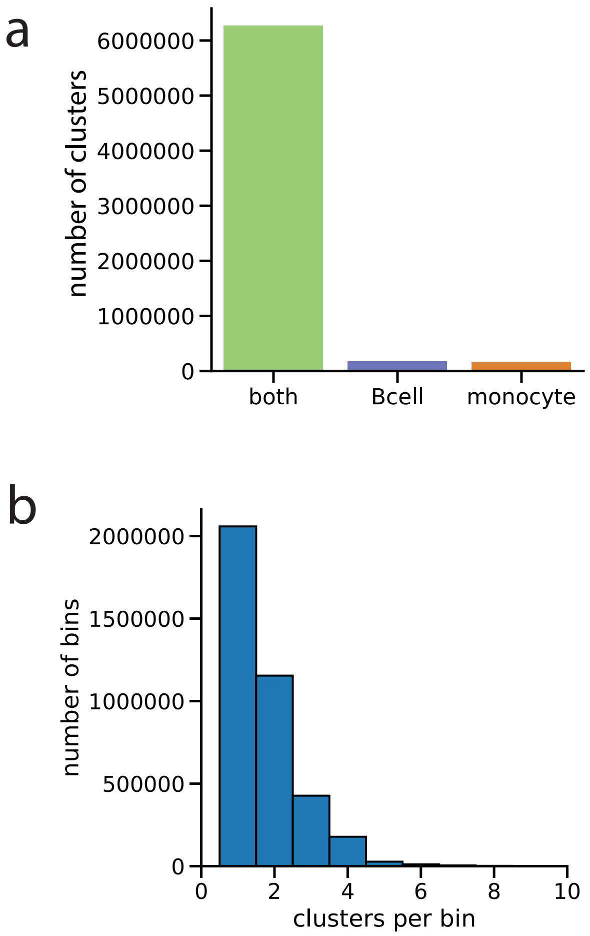

Supplementary Figure 2 – Genome-wide calculation of sample-specific CluBCpG clusters.
(**a**) Bar chart showing total numbers of clusters identified genome wide between ENCODE B cells and monocytes. Bars show total number of clusters found in both samples, B cell only, and monocyte only. (**b**) Histogram depicting the distribution of the number of clusters per bin from ENCODE B cells and monocytes; x-axis truncated at 10 for clarity.

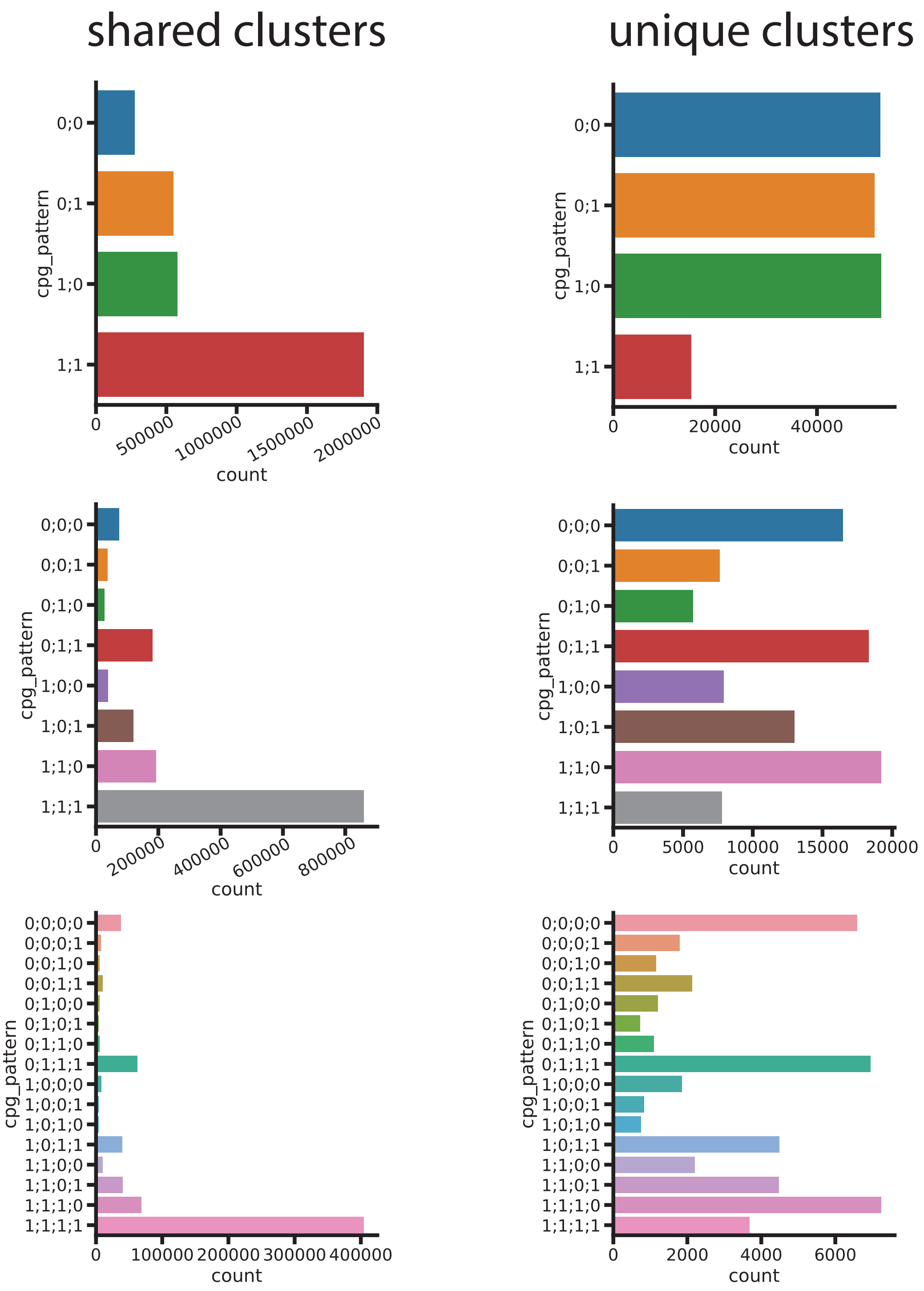

Supplementary Figure 3 – Predominant CpG patterns differ between shared and cell type-specific clusters
Bar plots showing the total counts of different CpG patterns identified across the full genome. Shared clusters (left) were clusters with patterns found in both B cells and monocytes. Unique clusters (right) were found only in one cell type. Plots are separated by CpG density (i.e. 2, 3, or 4 CpGs/bin). On the y-axes a number 1 indicates a methylated CpG site, 0 is unmethylated.

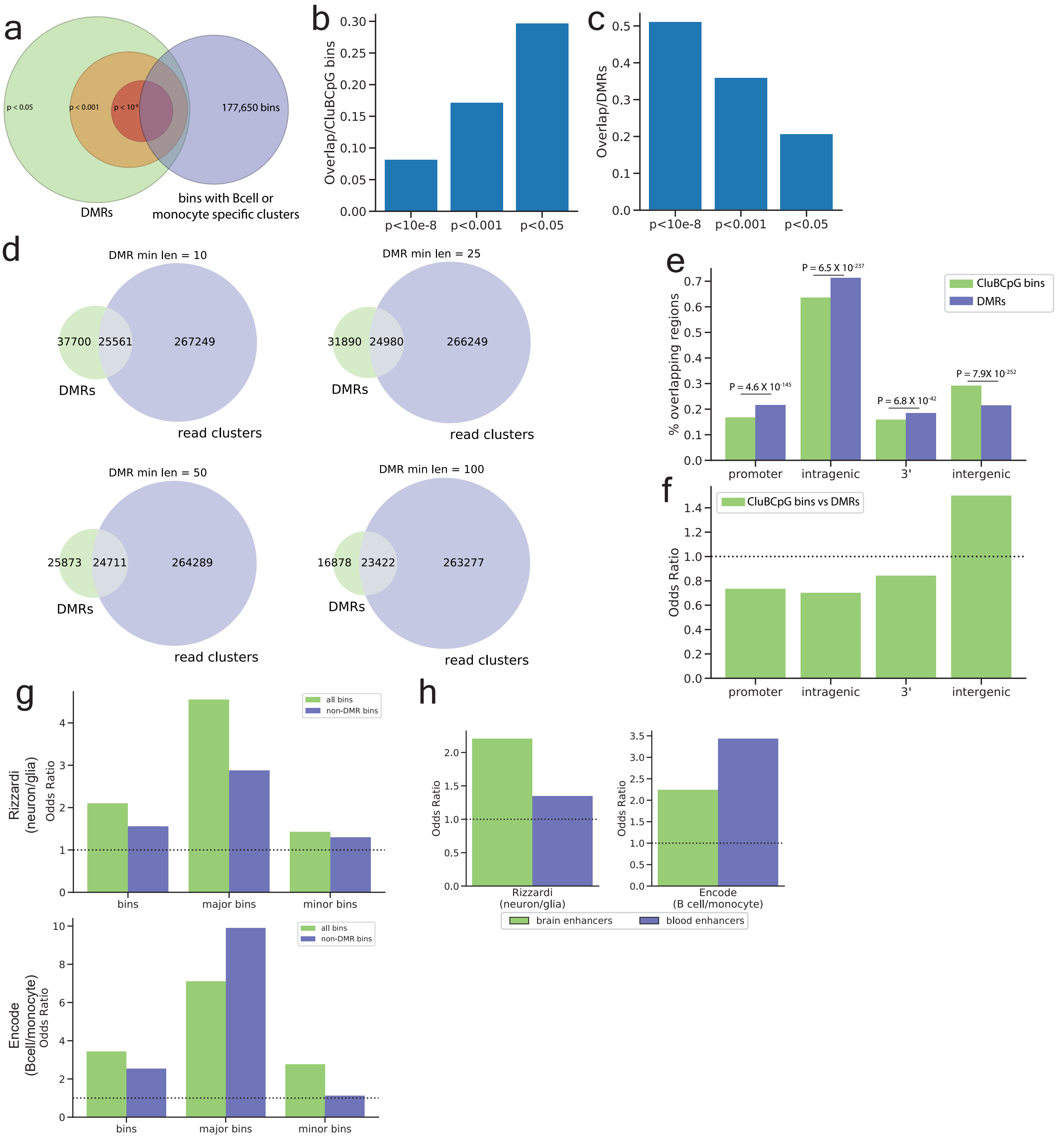

Supplementary Figure 4 – CluBCpG identified regions are predominantly found outside of DMRs.
(**a**) Venn diagram showing the overlap of CluBCpG-identified bins with read clusters and DSS-identified DMRs using different p-value thresholds. Size of circles scale with number of regions. (**b-c**) Bar plots showing the ratio of overlapping regions in (a) to (**b**) total bins with B cell or monocyte specific read clusters and (**c**) the ratio of overlapping regions to total DMRs. (**d**) Venn diagrams depicting the overlap between DMRs and bins when adjusting the minimum length threshold within DSS. (**e**) Bar plot showing the percentage of bins with a cell type-specific cluster (green) and DMRs (purple) overlapping annotated genic features. (**f**) Bar plot of the odds ratio calculated from the overlaps in (d); annotated genomic features are defined as: promoter=transcription start site (TSS) +/- 3kb; intragenic=TSS- transcription end site (TES); 3'=TES +/- 3kb; intergenic=all other genomic regions. (**g**) The odds ratio of the overlap between cell type-specific bins and enhancer regions. Non-DMR bins have had all bins overlapping a DMR removed from the analysis. (**h**) Odds ratio of the overlap between cell type-specific clusters and cell type-specific active enhancers.

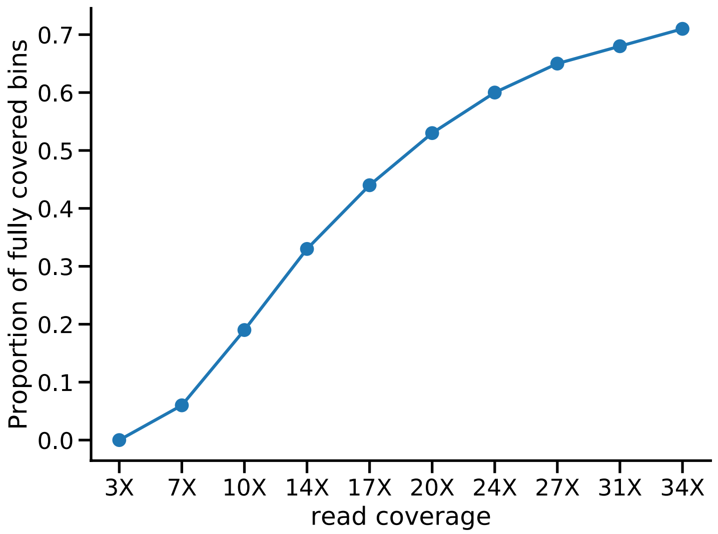

Supplementary Figure 5 – CluBCpG informative bins as a function of read coverage.
Proportion of bins with ≥ 10 fully covered reads vs. average read depth of the sequencing data. Calculations were performed on chromosome 19 from ENCODE B cells.

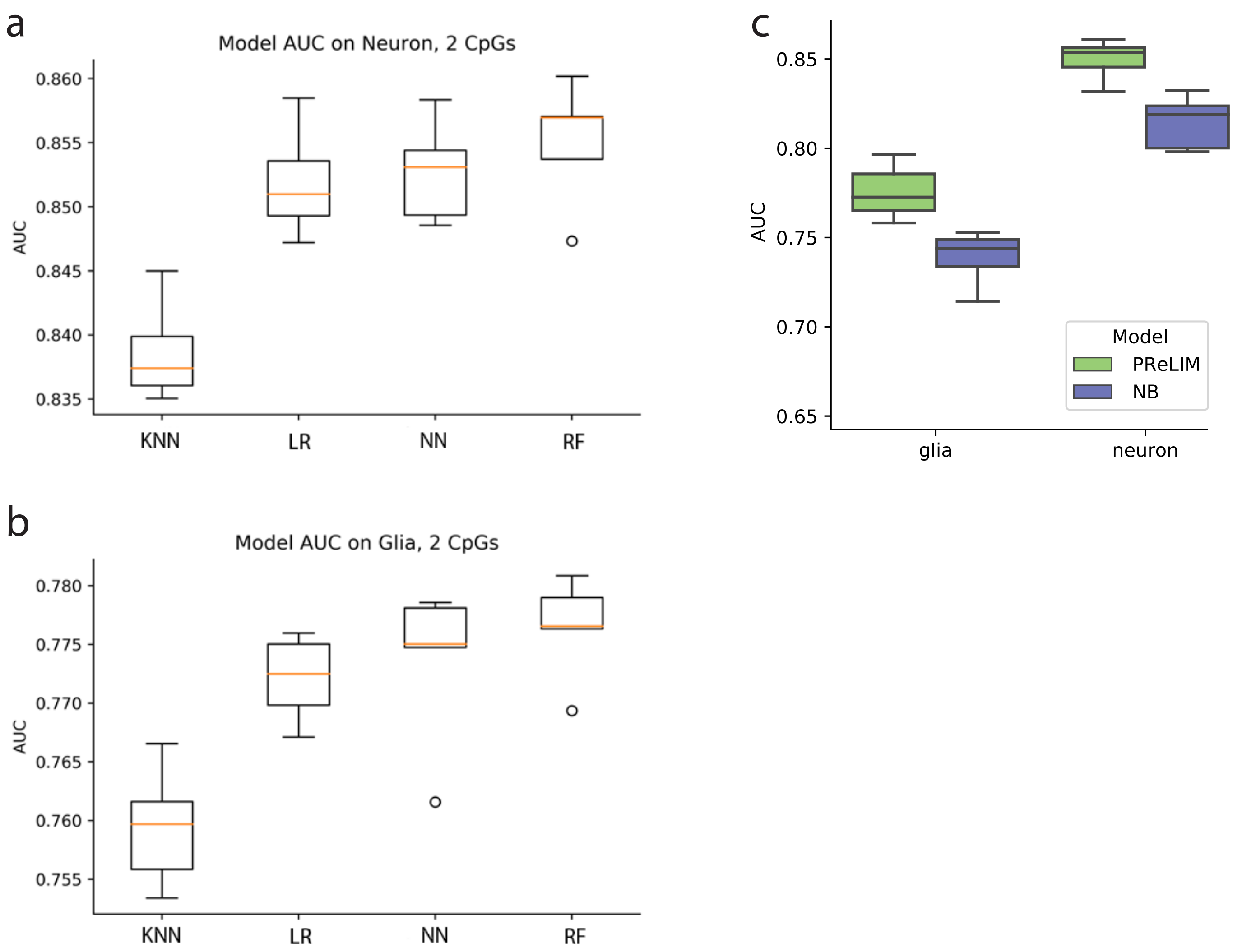

Supplementary Figure 6 – Comparison of multiple machine learning algorithms.
Box and whisker plots showing the area under the receiver operating characteristic curve (AUC) for imputation by multiple machine learning algorithms; KNN=K nearest neighbors, LR=logistic regression, NN=neural network, RF=random forest, NB=Naive Bayes. AUCs were calculated from 5-fold cross validation of each model on data from mouse neurons (**a**) and glia (**b**). (**c**) PReLIM was compared against a Naive Bayes model which uses only the Column mean (average methylation at each CpG site) as a feature. No confidence filtering was performed for these comparisons.

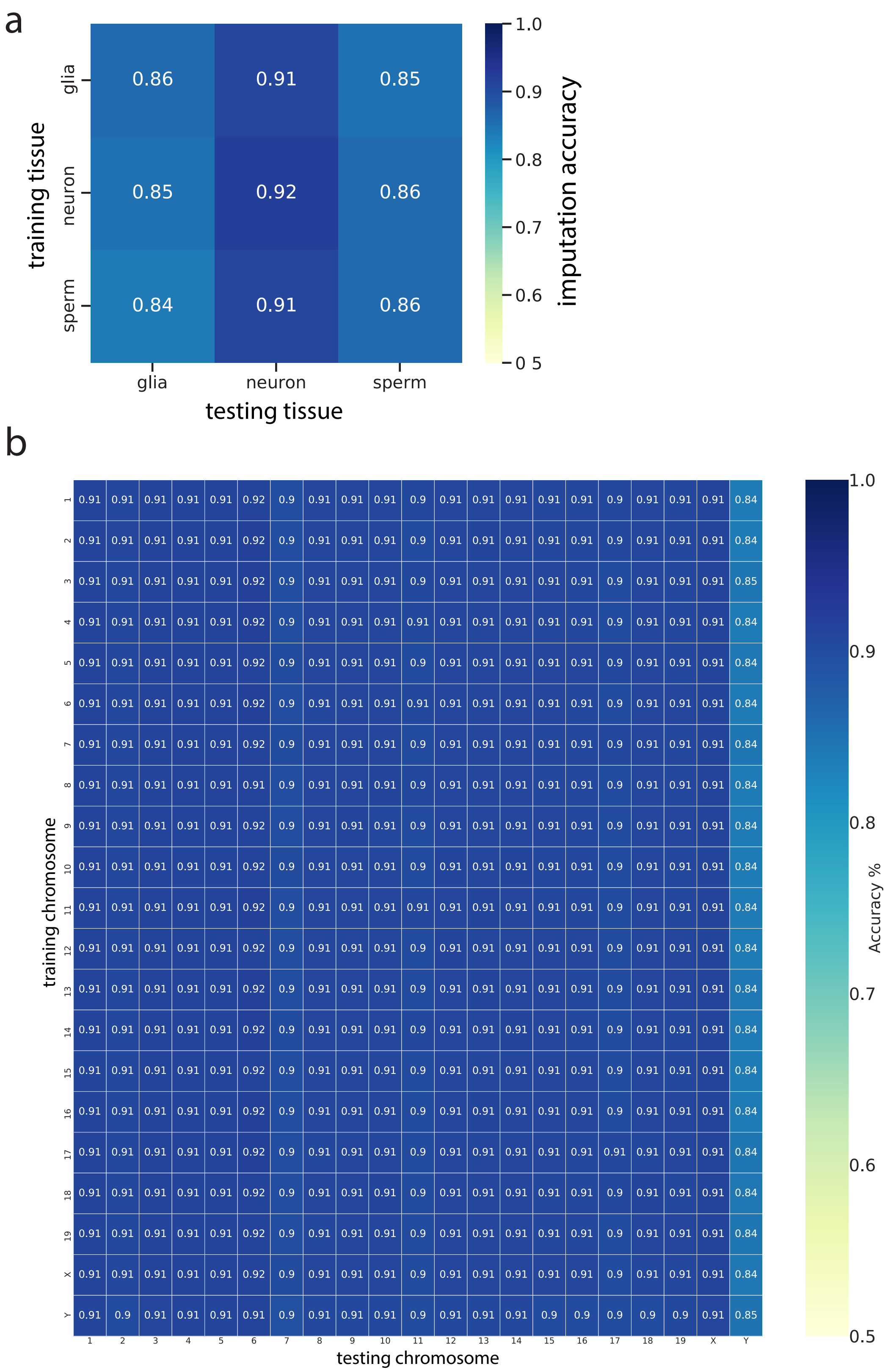

Supplementary Figure 7 – PReLIM cross-tissue and cross-chromosome performance
(**a**) Heatmap showing how a PReLIM model trained on one library performs when predicting on a different library. (**b**) Heatmap showing accuracy of PReLIM when trained on one mouse neuron chromosome and predicted on all other chromosomes.

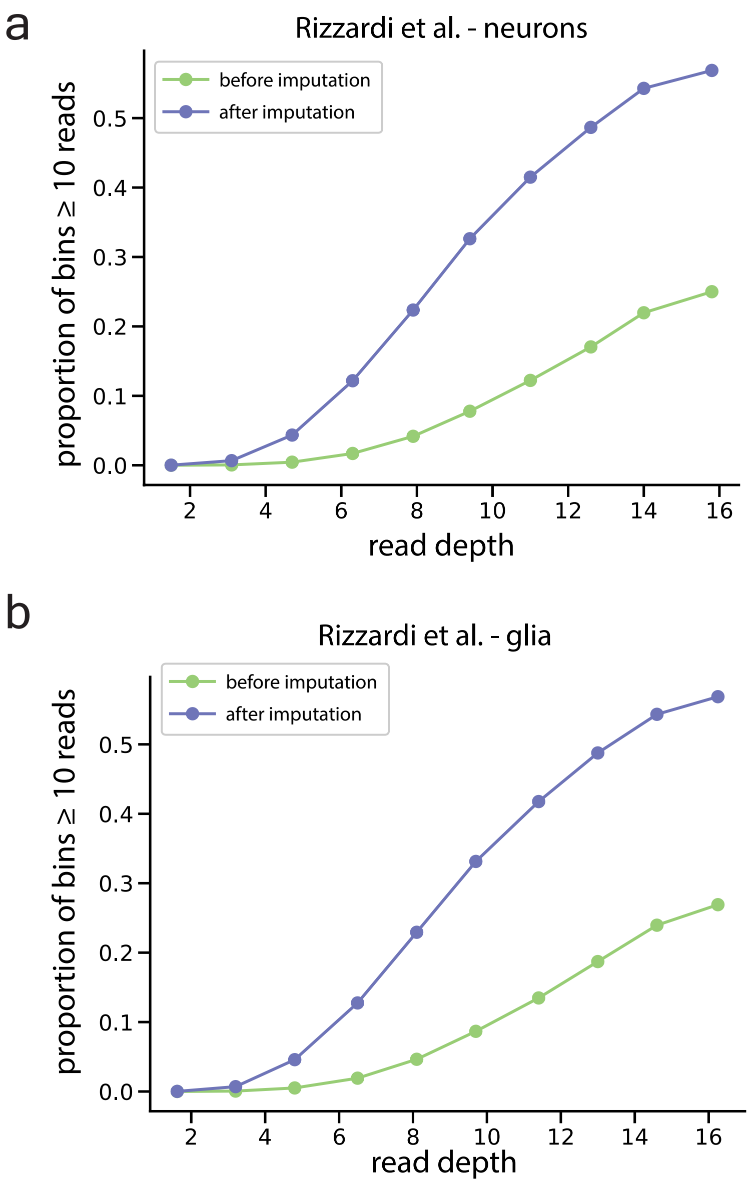

Supplementary Figure 8 – Imputation gains in informative bins vs. average sequencing depth.
(**a-b**) Line graphs showing the proportion of bins with ≥ 10 reads covering all CpGs before and after imputation with PRe-LIM on human (**a**) neurons and (**b**) glia. Calculations were performed using chromosome 19.

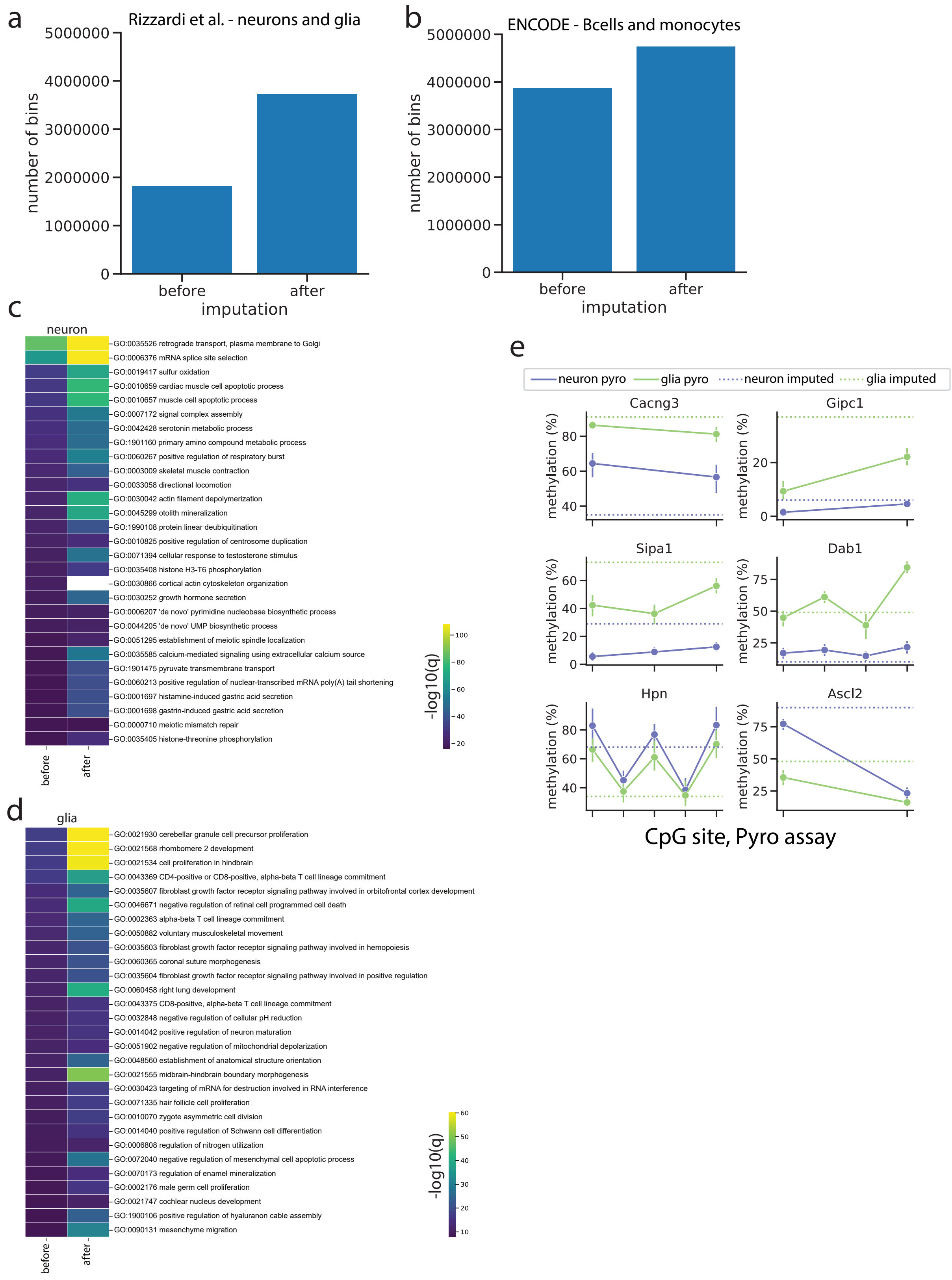

Supplementary Figure 9 – Genome-wide imputation gains in informative bins.
(**a-b**) Bar plots showing the number of bins with ≥10 reads genome-wide when analyzing human neuron and glia (**a**) and human B cells and monocytes (**b**) before and after imputation. (**c-d**) Heatmap showing the top 30 GO biological process terms with bins unique to either neuron (**c**) or glia (**d**) before and after imputation. Analysis was performed using GREAT, colors represent $-\log_{10}$ of the q-value. (**e**) Pyrosequencing plots of novel DMRs found only post-imputation using PReLIM on the mouse neuron and glia WGBS data. Each point represents one CpG site and are connected by a line. Horizontal dotted lines indicate average cell type-specific methylation across the DMR, from the WGBS data following PReLIM imputation.

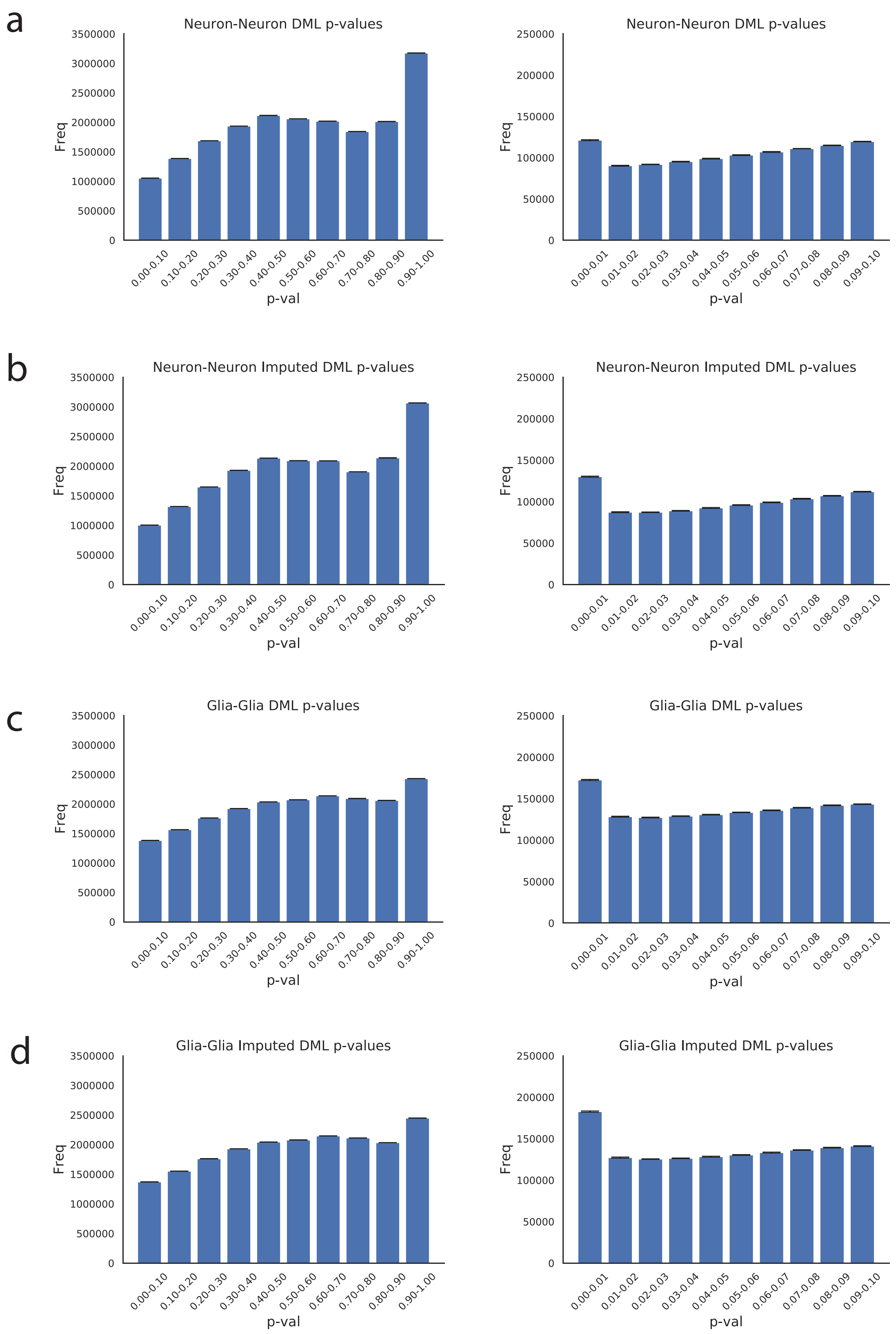

Supplementary Figure 10 – No effect of imputation on DSS P-value distribution under the null.
P value distributions for differentially methylated locus (DML) test statistic from DSS, in self vs. self comparisons before and after imputation by PReLIM. Each histogram shows the mean and standard deviation (error bars) across 10 random 50:50 splits of WGBS reads from mouse neuron or glia from [27]. In each panel, the full P value distribution (0-1) is shown on the left and the low range (0-0.1) on the right. (**a**) Neuron vs. neuron. (**b**) Neuron vs. neuron following imputation by PReLIM. (**c**) Glia vs. glia. (**d**) Glia vs. glia following imputation by PReLIM.

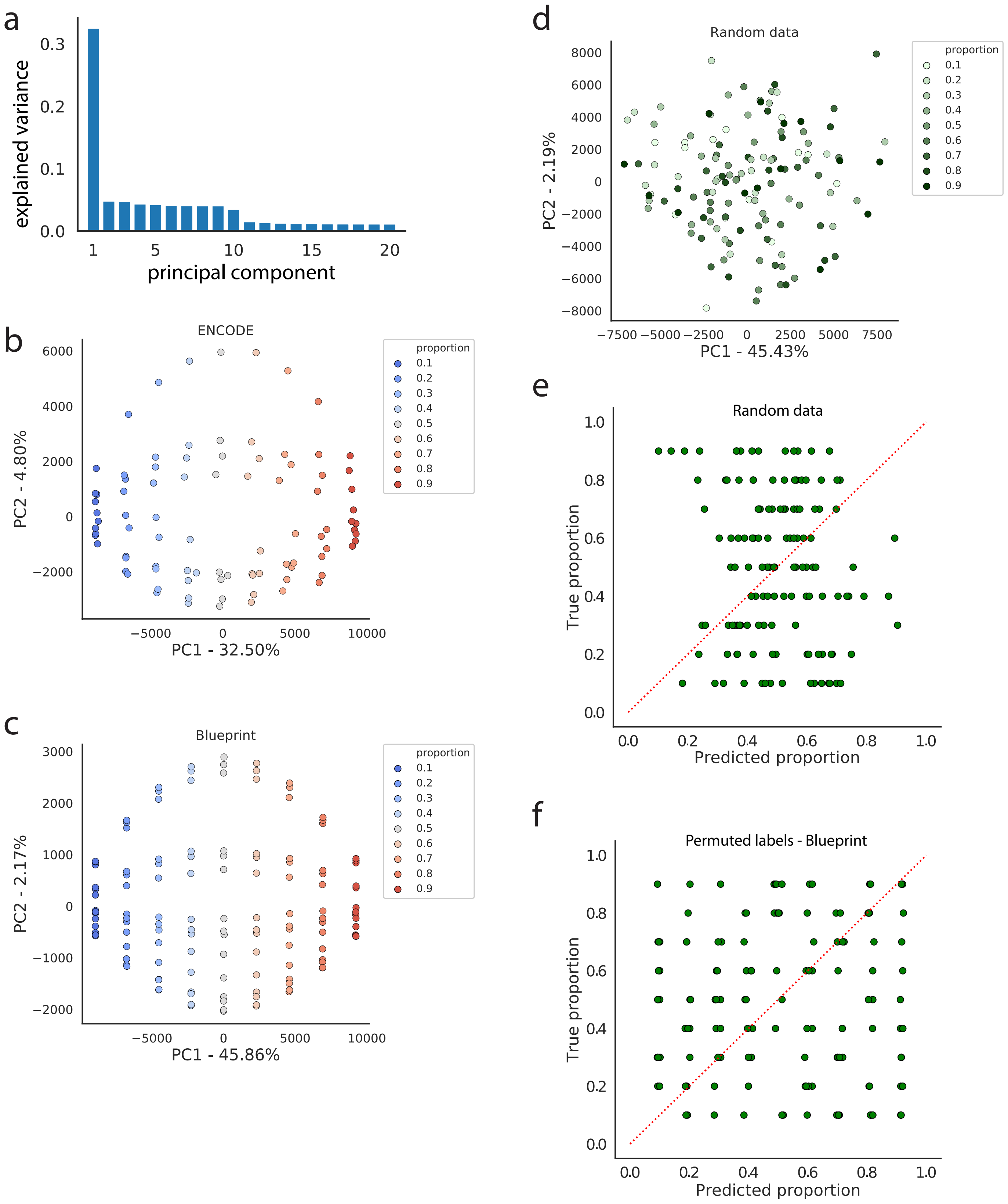

Supplementary Figure 11 – Deconvolution PCA and permutation analyses.
(**a**) Scree plot with bars showing proportion of explained variance provided by each of the 20 principal components (PCs). (**b-c**) Scatter plot of PC1 vs PC2 of the B cell:monocyte synthetic mixtures from ENCODE (**b**) and Blueprint (**c**). Colors represents the proportion of the mixture. (**d**) Scatter plot of PC1 vs PC2 on randomly generated data. (**e-f**) The predicted proportion vs true proportion on the randomly generated data (**e**) and Blueprint data with permuted proportion labels (**f**).

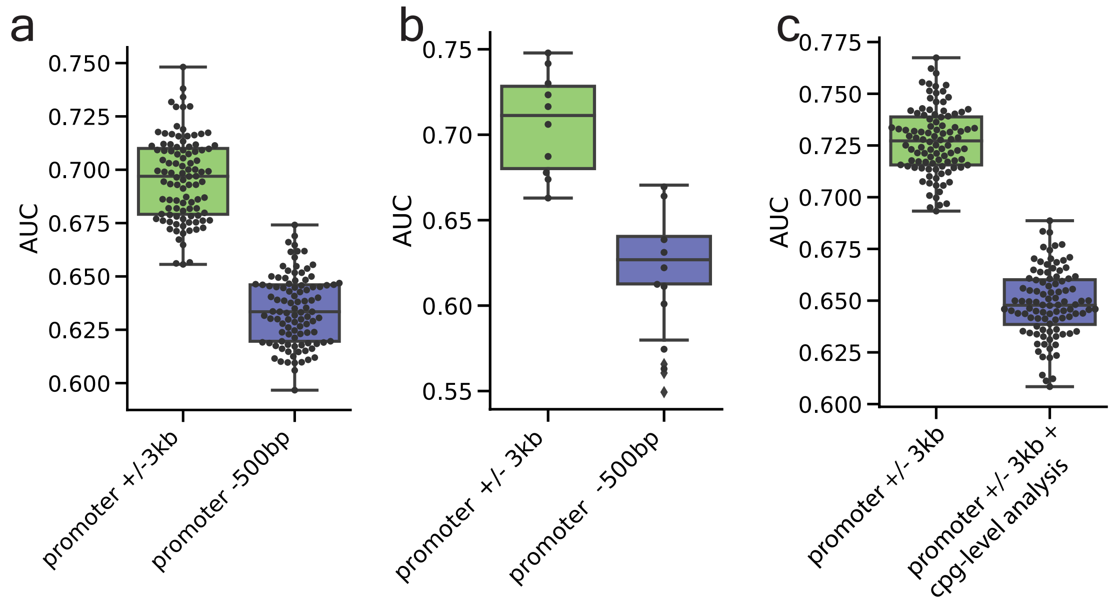

Supplementary Figure 12 – Evaluating alternate strategies to predict gene expression from promoter methylation. Random forest models using methylation data from promoter windows of +/- 3kb from the transcription start site (TSS) (green) and 500bp upstream of the TSS (purple) were compared against each other. Box and whisker plots overlaid with individual points showing the area under the ROC curve (AUC) for 100 random train-test splits (**a**) and 10-fold nested cross validation (**b**). To test the effect of including methylation levels at each of the individual CpG sites, the +/- 3k promoter window was broken down into methylation frequencies in decile blocks at the individual CpG-level and tested using 100 random train-test splits (**c**). Whiskers extend to 1.5x the interquartile range.
