# Supplementary material for: Identification of cell type-specific methylation signals in bulk whole genome bisulfite sequencing data
Source: Genome Biol. 2020 Jul 1;21:156. doi: 10.1186/s13059-020-02065-5 (PMC7329512; doi:10.1186/s13059-020-02065-5)
Supplement: Supplementary file 6 — Additional file 6. Review History. [file 13059_2020_2065_MOESM6_ESM.docx]

**Review History**

**First round of review:**

**Reviewer 1**

The authors describe an analysis of methylation cell type specific data (i.e., sorted data), and show that there are clusters of methylation patterns that are specific to cell-types. They further provide a random forest based algorithm for the imputation of methylation levels, and they describe the usage of their findings in a few downstream analyses, including estimation of gene expression, and the estimation of cell counts. Generally, I find the paper interesting, and I have the following major concerns/comments:

1. Innovation - I was under the impression that this phenomenon has been previously shown, however I am not sure what would be the correct reference, and it may just be an urbanic legend, and that the authors of this paper are the first ones to actually show an empirical evidence for the phenomenon. I refer to the other reviewers who may have complementary expertise to mine and would be familiar with the literature in order to know if this has already been shown as a paper or as part of another analysis.

2. Confounders - Even though the results are statistically significant, I am quite worried about the potential effects of biological and technical confounders on the results, since the analysis has been done on single samples (or at least the number of samples is not given so I assume it is either 1 or small). Imagine, for example, that the monocytes were taken from old people and the B cells were taken from young people. This will affect differences in methylation across the entire genome. The authors do provide some evidence for robustness of their results by running the analysis on another dataset, but these are only two datasets and they may share many of the biological confounders such as age, sex, etc. I think that more than a couple of datasets are needed for this analysis, and that some treatment of confounders using regression should be applied.

3. Imputation analysis - I am not convinced that the imputation analysis is sound. The authors show that 41% additional DMRs were discovered (line 229), but it's unclear that the imputation does not create inflation under the null. It'd be good to test two datasets with the same cell type and see whether there is also "improvement" in DMR detection there, so use this as a control experiment, and ideally that should be done under the same coverage conditions.

4. For the cell mixtures, in line 245, it is unclear from the text whether the same samples were used for the mixture and for training. If that's the case, this is problematic and the results are overfitted. Also, that section is not completely clear - is the regression performed on the PCs? Finally, the authors claim that the replication on another dataset excludes the option of confounders, but as I explained above, this is not true for biological confounders such as age and sex since those may have the same effect in both datasets.

5.For the gene expression section, I believe that the baseline is too simple and doesn't represent the contribution of the clusters to the AUC. I think that the baseline should include the methylation levels at each of the methylation sites (i.e., marginal frequencies and not the clusters). Otherwise it is unclear whether the clusters contributed anything.

**Reviewer 2**

This an interesting paper which introduces a very promising and I think quite novel method for the analysis of whole genome bisulfite sequencing data.  The method uses multiple CpG sites on a single sequence read to derive something akin to a haplotype which they hypothesise could function as a cell-type signature.  I think their analyses of public data sets derived from different cell types does succeed in showing that this is the case and that their method generates information that is different from the conventional DMR methods.  They also develop an imputation method which they show can recover additional information from low coverage bisulfite sequencing.  The software they have developed for these two methods is available on github.  I have not tested it myself but it looks well developed and I am very impressed by the quality of the documentation.

I think the biological insights offered by the CluBCpG method are likely to be important, and the authors have presented their reasoning, methods and results very clearly and with what looks like usable software.

**Authors’ response to reviewers**

We very much appreciate the detailed and broadly favorable comments from two expert referees. In response to the reviews we performed extensive additional analyses, the results of which strengthened our major claims.

In our manuscript, the applications of our read-level analysis software (CluBCpG) focus exclusively on single sample or two-sample comparisons of whole-genome bisulfite sequencing (WGBS) libraries. Since our original submission, we scaled-up and began applying CluBCpG to pools of WGBS profiles comprising dozens of samples. During that process we identified a technical scalability limitation in that ClubCpG was not handling conflicts of reads with the same name originating from different WGBS libraries. We investigated and solved this issue, and the changes are reflected in the latest version of CluBCpG downloadable from GitHub and Zenodo. We repeated all CluBCpG analyses in the manuscript using this latest version. This resulted in minor changes in numbers of covered bins (e.g. lines 115 and 164), but did not substantively change the results or

our interpretation. Although this refinement contributed to a delay in the resubmission of our manuscript, we made this extra effort because the greatly improved scalability and user experience will lead to increased widespread adoption of the ClubCpG software.

**REVIEWER 1**

**Comment**: The authors describe an analysis of methylation cell type specific data (i.e., sorted data), and show that there are clusters of methylation patterns that are specific to cell-types. They further provide a random forest based algorithm for the imputation of methylation levels, and they describe the usage of their findings in a few downstream analyses, including estimation of gene expression, and the estimation of cell counts. Generally, I find the paper interesting, and I have the following major concerns/comments.

**Response**: We are pleased that the reviewer finds our paper interesting.

**Comment**: 1. Innovation - I was under the impression that this phenomenon has been previously shown, however I am not sure what would be the correct reference, and it may just be an urbanic legend, and that the authors of this paper are the first ones to actually show an empirical evidence for the phenomenon. I refer to the other reviewers who may have complementary expertise to mine and would be familiar with the literature in order to know if this has already been shown as a paper or as part of another analysis.

***Response****: Before drafting our original manuscript (indeed, before embarking on development of this new approach) we conducted a detailed literature search to determine if previous groups had successfully mined read-level patterns of CpG methylation to draw inferences regarding different cell types in heterogeneous tissues. Relevant literature identified in our review was described in the Introduction of our original manuscript. In response to the reviewer’s comment, however, we updated and expanded our search on PubMed, usingpermutations of the following search terms:*

*read level*

*whole genome bisulfite sequencing read level analysis*

*WGBS AND cell type or cell types*

*bisulfite sequencing cell type specific*

*cell subset specific*

*This search identified two additional relevant publications (Sun et al 2016 Scientific Reports and Arai et al 2016 PLOS One). We now cite these in the Introduction (Lines 69-72):*

*“Other attempts at exploring read-level information in WGBS data sets have focused on minority regions such as those exhibiting ‘bipolar methylation’ [13] or containing ‘hypo-methylated alleles’ [14], rather than assessing the full breadth and depth of potential cell type-specific signals.”*

*Importantly, in no way do these previous papers undermine the innovation of our manuscript.*

**Comment**: 2. Confounders - Even though the results are statistically significant, I am quite worried about the potential effects of biological and technical confounders on the results, since the analysis has been done on single samples (or at least the number of samples is not given so I assume it is either 1 or small). Imagine, for example, that the monocytes were taken from old people and the B cells were taken from young people. This will affect differences in methylation across the entire genome. The authors do provide some evidence for robustness of their results by running the analysis on another dataset, but these are only two datasets and they may share many of the biological confounders such as age, sex, etc. I think that more than a couple of

datasets are needed for this analysis, and that some treatment of confounders using regression should be applied.

***Response****: We thank the reviewer for pointing out this alternative explanation for our findings. Regarding the suggestion that more than a couple of data sets are needed for this analysis, we searched the literature and could not find additional WGBS data sets on the human sorted cell types we studied. However, owing to the characteristics of the data sets studied, our results cannot be explained by confounding by donor sex or age. In response to the reviewer’s comments, we have included an additional supplemental table (Supplemental Table 3), detailing subject data on the ENCODE and Blueprint B cell and monocyte samples. The issue of potential confounding by age and sex is now addressed in two places. We bring it up in our initial analysis of cell type specific information in the read clusters (Lines 107-109):*

*“Importantly, the B cells and monocytes were isolated from one individual (a 37 year old male)*

*(Supplementary Table 3), and the neuron and glia samples were likewise isolated from one individual (a 40 year old male), ruling out potential confounding by age, sex, etc.”*

*We also now address this issue in the section detailing our ability to deconvolute synthetic mixtures of B cells and monocytes (Lines 270-273):*

*“Note that the ENCODE B cell and monocyte data are from one (37 year old male) individual, and the Blueprint data are from at least one female (65-70 years old) (Supplementary Table 3); hence, the agreement of these models cannot be explained by confounders such as age or sex.”*

**Comment**: 3. Imputation analysis - I am not convinced that the imputation analysis is sound. The authors show that 41% additional DMRs were discovered (line 229), but it's unclear that the imputation does not create inflation under the null. It'd be good to test two datasets with the same cell type and see whether there is also "improvement" in DMR detection there, so use this as a control experiment, and ideally that should be done under the same coverage conditions.

***Response****: We thank the reviewer for proposing this alternative explanation. However, if PReLIM artifactually caused inflation under the null, then many DMRs identified following PReLIM would not validate. Hence our independent validation of 10 out of 10 DMRs identified only after applying PReLIM (Figure 4e and Supplementary Figure 9e) argues strongly against the reviewer's concern. Nonetheless, in response to this concern we conducted a new analysis, performing 10 random 50:50 splits of both the neuron and glia WGBS reads and evaluating the CpG-level P value distribution by DSS before and after imputation. The results (Supplementary Figure 10) show that PReLIM introduces no inflation under the null. In addition to this new supplementary figure, this new analysis is now described under Results (Lines 237-240):*

*“As a negative control, we generated multiple splits and evaluated the CpG-specific P value distribution from DSS for neuron vs. neuron and glia vs. glia, before and after imputation. The results (Supplementary Figure 10) show that PReLIM does not introduce inflation under the null.”*

**Comment**: 4. For the cell mixtures, in line 245, it is unclear from the text whether the same samples were used for the mixture and for training. If that's the case, this is problematic and the results are overfitted. Also, that section is not completely clear - is the regression performed on the PCs? Finally, the authors claim that the replication on another dataset excludes the option of confounders, but as I explained above, this is not true for biological confounders such as age and sex since those may have the same effect in both datasets.

***Response****: We thank the reviewer for pointing out that our description was not perfectly clear. We now make it explicit that the top 20 components from the PCA are used in the regression model, and now describe the meaning of "five-fold cross validation", to clarify that in each case testing was performed on the 20% of the data that was not used for training, so the models are not overfitted (Lines 261-265):*

*“We performed dimensionality reduction using principal component analysis (PCA) and kept the top 20 components for use in a multivariate linear regression model (Supplementary Figure 11a-b). To verify our model is not overfitting, we carried out 5-fold cross-validation, consistently obtaining a root mean squared error (RMSE) of 0.001 (Figure 5c) on each testing set which was held back from training.”*

*We addressed the issue of potential confounding by age and sex in response to comment #2, above.*

**Comment**: 5.For the gene expression section, I believe that the baseline is too simple and doesn't represent the contribution of the clusters to the AUC. I think that the baseline should include the methylation levels at each of the methylation sites (i.e., marginal frequencies and not the clusters). Otherwise it is unclear whether the clusters contributed anything.

***Response****: This is a great suggestion. We hadn’t thought of this, but agree it is important to determine if including CpG-level methylation information within the promoter improves the ability to predict gene expression. In other words, is it really the clusters, or simply the information in the individual CpGs? To test this, we built a new model based on marginal methylation frequencies at all CpGs within each promoter, and provide the results in a new supplementary figure (Supplementary Figure 12c). Remarkably, compared to promoter-average methylation, including this CpG-level model actually decreased the ability to predict gene expression. Hence, this new analysis strengthens our claim that the clusters themselves carry epigenetic information. We have included a new paragraph in Results describing this analysis (Lines 306-312):*

*“We wished to test whether the improved ability to predict expression is inherent to the methylation patterns within the clusters, or simply reflects the inclusion of CpG-level data. We therefore calculated the frequencies of CpG methylation-deciles for each gene promoter and included these in the baseline random forests model (Supplementary Table 4). Surprisingly, the inclusion of these data decreased the performance of the model (AUC 0.65 vs. 0.73) (Supplementary Figure 12c), indicating that the cluster pattern information does indeed contain cell-type specific information beyond that in the individual CpG methylation levels.”*

**REVIEWER 2**

**Comment**: This an interesting paper which introduces a very promising and I think quite novel method for the analysis of whole genome bisulfite sequencing data. The method uses multiple CpG sites on a single sequence read to derive something akin to a haplotype which they hypothesise could function as a cell-type signature. I think their analyses of public data sets derived from different cell types does succeed in showing that this is the case and that their method generates information that is different from the conventional DMR methods. They also develop an imputation method which they show can recover additional information from low coverage bisulfite sequencing. The software they have developed for these two methods is available on github. I have not tested it myself but it looks well developed and I am very impressed by the quality of the

documentation. I think the biological insights offered by the CluBCpG method are likely to be important, and the authors have presented their reasoning, methods and results very clearly and with what looks like usable software.

***Response****: We very much appreciate these many strongly favorable comments.*

**Second round of review**

**Reviewer 1**

The authors provide adequate responses to the major comments. There is only one minor issue that remains, regarding confounders: There still may. be technical confounders that create different patterns between different cell types. This may not be critical since this is replicable, but it would be good to acknowledge this in case researchers would like to apply these insights to other technologies.

**Reviewer 2**

The other reviewer was more skeptical than I was and had questions, some of which were well founded checks on the possibility that the results could be the result of confounders or overfitting.  The authors have gone through and made responses to all of the reviewer comments, making changes to manuscript for each one.  I think the authors have done the questions justice and have further improved the manuscript.
